# Innovative Hydrogel Design: Tailoring Immunomodulation for Optimal Chronic Wound Recovery

**DOI:** 10.1002/advs.202412360

**Published:** 2024-11-22

**Authors:** Chun‐Mei Lai, Wei‐Ji Chen, Yuan Qin, Di Xu, Yue‐Kun Lai, Shao‐Hua He

**Affiliations:** ^1^ College of Life Sciences Fujian Provincial Key laboratory of Haixia applied plant systems biology Fujian Agriculture and Forestry University Fuzhou Fujian 350002 P. R. China; ^2^ Shengli Clinical Medical College of Fujian Medical University Department of Pediatrics surgery, Fujian Provincial Hospital University Affiliated Provincial Hospital, Fuzhou University Affiliated Provincial Hospital 134 Dongjie Road Fuzhou Fujian 350001 P. R. China; ^3^ National Engineering Research Center of Chemical Fertilizer Catalyst (NERC‐CFC) College of Chemical Engineering Fuzhou University Fuzhou 350116 P. R. China

**Keywords:** chronic wounds, immunomodulatory hydrogel, macrophage polarization, tissue

## Abstract

Despite significant progress in tissue engineering, the full regeneration of chronic wounds persists as a major challenge, with the immune response to tissue damage being a key determinant of the healing process's quality and duration. Post‐injury, a crucial aspect is the transition of macrophages from a pro‐inflammatory state to an anti‐inflammatory. Thus, this alteration in macrophage polarization presents an enticing avenue within the realm of regenerative medicine. Recent advancements have entailed the integration of a myriad of cellular and molecular signals into hydrogel‐based constructs, enabling the fine‐tuning of immune cell activities during different phases. This discussion explores modern insights into immune cell roles in skin regeneration, underscoring the key role of immune modulation in amplifying the overall efficacy of wounds. Moreover, a comprehensive review is presented on the latest sophisticated technologies employed in the design of immunomodulatory hydrogels to regulate macrophage polarization. Furthermore, the deliberate design of hydrogels to deliver targeted immune stimulation through manipulation of chemistry and cell integration is also emphasized. Moreover, an overview is provided regarding the influence of hydrogel properties on immune traits and tissue regeneration process. Conclusively, the accent is on forthcoming pathways directed toward modulating immune responses in the milieu of chronic healing.

## Graphical abstract

Here, it is summarized advanced technologies for rationally designing immunomodulatory hydrogels to control macrophage polarization, including controlled immune stimulation through hydrogel chemistry and cell/molecules integration, as well as surface modification, reviewing the influence of hydrogel properties on immunogenic features and chronic wound recovery, the evolving future directions and emerging research strategies for immune response control in chronic wound healing also highlighted.

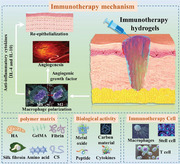

## 1 Introduction

The skin, revered as the crucial organ in the human body, functions as a robust outer shield that defends internal organs against external dangers. It activates a multifaceted array of immune defense responses to combat a range of external stimuli, encompassing mechanical, chemical, and pathogenic microorganisms. Structurally, the skin's intricate structure comprises diverse layers housing a plethora of immune and non‐immune cells, notably the outer epidermis and the underlying dermis, intertwined with a sophisticated vascular. Furthermore, there exists a subcutaneous layer known as the hypodermis, which provides support to the overlying layers and is mainly comprised of adipose tissue and connective tissues.^[^ ^1^ ^]^ Within the epidermis, where keratinocytes are located, there is an active defense against infectious microorganisms and a regulation of body hydration, while the dermis provides structural support to the skin and delivers essential nutrients for maintaining epidermal equilibrium. Various cell types in the epidermis carry out specific roles that collectively contribute to its overall function. Diverse specialized cell types within the epidermis perform distinct functions that synergistically contribute to its integrity. For example, Melanocytes contribute to skin pigmentation, Merkel cells serve as mechanoreceptors through intimate interactions with sensory neurons, and Langerhans cells (LCs) play a vital role as antigen‐present dendritic cells (DCs).^[^ ^2^ ^]^ Moreover, the dermis layer is abundant in collagen protein and contains stromal cells such as fibroblasts, human dermal microvascular endothelial cells (HDMECs), and pericytes. Furthermore, the dermis layer exhibits a robust vascular network comprising blood and lymphatic vessels, establishing a connection to the epidermis through the membrane.^[^ ^3^ ^]^ The skin's cellular populace, intricately scattered throughout its tri‐layered architecture, orchestrates a multifaceted symphony of structural reinforcement and immunological fortification. Remarkably, a sophisticated interplay among these cells orchestrates a synchronized immune defense mechanism within the skin, amplifying its protective prowess. Noteworthy are keratinocytes, melanocytes, fibroblasts, and endothelial cells‐non‐traditional immune players‐exerting indispensable influence in inflammation modulation and immune response coordination, while concurrently serving as indispensable structural constituents of skin.^[^ ^4^ ^]^

Considering that the skin is the first line of defense against the environment, it is susceptible to disruption from various sources such as trauma, injury, burns, ulcers, surgical procedures, chronic illnesses, and inflammatory skin reactions. These disruptions to the skin's epithelial and connective tissue structures often impair its fundamental functions, necessitating a reparative process known as healing.^[^ ^5^ ^]^ The wound healing process is intricately orchestrated, progressing through well‐defined stages of hemostasis, inflammation, proliferation, and remodeling. In instances of chronic wound healing, this sequential progression is disrupted, presenting as persistent, low‐grade inflammation that is theorized to impede or prolong the wound healing.^[^ ^6^ ^]^ The initial phase of this process involves the constriction of blood vessels and the aggregation of platelets at the injury site to cease bleeding, followed by the initiation of a blood clot through fibrin strands. Concurrently, the wound healing mechanism encompasses swift innate immune responses that identify damaged cells, pathogens, and bacteria through the activity of white blood cells, complemented by slower adaptive immune responses for pathogen eradication. Subsequently, the proliferative phase commences, leading to the formation of granulation tissue, re‐epithelialization, and neovascularization over the ensuing weeks. This phase facilitates wound contraction and the establishment of new tissue with a vascular network to ensure optimal oxygen and nutrient delivery. Finally, during the remodeling phase, the matrix and cells undergo reorganization to restore normal tissue architecture or generate mature scars.^[^ ^7^ ^]^ Chronic wounds typically result from extensive tissue damage that impedes the healing process and are often coupled with infections as well as the buildup of microbial biofilms. Prompt and effective intervention is essential for these wounds, as neglecting to treat them can lead to serious complications such as limb amputation, systemic sepsis, and in the most severe cases, can be fatal.^[^ ^8^ ^]^

In recent decades, many approaches to chronic wound care have been extensively researched, focusing on different stages of the healing cycle.^[^ ^9^ ^]^ The current clinical guidelines emphasize the importance of initial and routine debridement to thoroughly remove devitalized tissue and adjacent callus material. Additionally, recommendations include the utilization of wound dressings, such as moist gauze, to create an optimal wound environment, absorb excess exudate, prevent maceration of surrounding skin, administer cytokines and growth factors, implement cell therapy, and applying electrical or mechanical stimulations.^[^ ^10^ ^]^ These dressings serving as interim agents to bolster the wound healing process, are hailed as the pinnacle of treatment efficacy. Consequently, a new generation of biomaterials‐driven wound dressings has emerged, designed to mimic the intricate skin microenvironment. Among these strategies, the innovative artificial constructs, serving as interim agents to bolster the wound healing process, are hailed as the pinnacle of treatment efficacy. Consequently, a new generation of biomaterials‐driven wound dressings has emerged, designed to mimic the intricate skin microenvironment.^[^ ^11^ ^]^ Among all these biomaterials, hydrogels, characterized by their biomimetic structures and tactile properties, have been extensive used in wound care.^[^ ^12^ ^]^ Due to their exceptional biocompatibility and ability to encapsulate a range of cells and bio‐macromolecules, hydrogels have emerged as a potential option for wound healing. Their potential is further enhanced by their ability to provide controlled release in response to various external stimuli. These adaptable systems enable targeted and prolonged release of immunomodulatory agents and cellular components, increasing treatment efficacy and reducing the risk of systemic adverse reactions. Furthermore, hydrogels can be engineered to closely emulate the natural 3D architecture of the extracellular matrix (ECM), offering the necessary biophysical and biochemical cues to attract and activate endogenous immune cells, thereby facilitating a range of therapeutic tasks.^[^ ^13^ ^]^

Specific ingredients such as various nanomaterials, growth factors, cytokines, and hydrogels can effectively stimulate each phase of the wound healing process.^[^ ^11^, ^14^ ^]^ Currently, numerous recent review articles have delved into the latest advancements and contemporary clinical methodologies that bolster wound healing, offering enhanced treatment options for individuals grappling with chronic wounds.^[^ ^15^ ^]^ The role of the immune system in the chronic wound healing trajectory has spurred considerable interest in the development of varied immunomodulatory therapeutic strategies within the realm of current biomedical research. These strategies aim to regulate immune responses post‐injury, fostering an anti‐inflammatory milieu that expedites the wound healing process. Notably, recent literature has underscored the vital significance of immunomodulation strategies in steering tissue regeneration.^[^ ^11^ ^]^

An exemplary illustration of this can be found in the research conducted by Julier et al. Their work illuminates a diverse array of biomaterials engineered for the targeted delivery of stem cells and pharmaceutical agents.^[^ ^16^ ^]^ These innovative materials not only facilitate superior tissue regeneration but also exhibit a remarkable capacity to attenuate fibrous formation. Furthermore, in a separate review article, Larouche and collaborators have succinctly summarized a myriad of approaches honed in on the pathophysiology of acute and chronic wounds, aimed at modulating the immune system to hasten the healing cascade.^[^ ^17^ ^]^ Despite the wealth of research on immunomodulatory strategies involving hydrogels, our current comprehension indicates that the utilization of these biomaterials for the enhancement of wound healing remains an area yet to be investigated.

**Figure 1:**
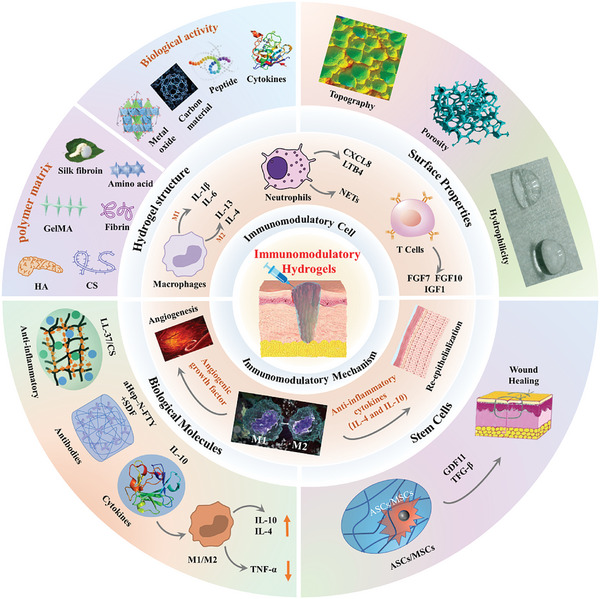
A visual odyssey into hydrogel‐driven immunomodulation, showcasing a spectrum of advanced strategies for therapeutic innovation.

In this review, we will provide an overview of cutting‐edge approaches employed to accelerate the healing of chronic wounds using various immunomodulatory hydrogels. Initially, an exploration of various skin conditions and their corresponding immune characteristics will be conducted. Subsequently, the focus will shift toward the latest advancements in the treatment of chronic wounds, emphasizing the potential of immunomodulation‐based approaches as the future of wound management. Following this, innovative immunomodulation techniques revolving around the chemical composition of hydrogel materials, surface attributes, and the integration of cells and bioactive agents will be scrutinized. **Figure** **1** provides a visual representation of these methodologies. Lastly, the discussion will encompass the potential clinical implications and future trajectories of advanced wound healing.

## 2 Deciphering Dermatological Pathologies: The Key Role of Immunity in Wound Regenesis

Based on the healing process timeline, skin injuries are categorized as either acute or chronic wounds.^[^ ^18^ ^]^ Acute wounds maintain the skin's integrity and progress through the standard stages of wound healing, whereas chronic wounds exhibit a deviation from the typical healing phases due to an imbalance in pro‐ and anti‐inflammatory signals, altering the normal skin responses.^[^ ^19^ ^]^ Currently, in China, ≈1 billion wound care treatments are required annually, with ≈30 million cases being classified as severe.^[^ ^20^ ^]^ Within chronic wounds, enduring inflammation results in the most disruption to the functionality of inflammatory cells, alongside the most substantial deviations in cytokine and growth factor concentrations as opposed to standard wounds.^[^ ^21^ ^]^ These adaptations are correlated with the elevated levels of inflammation, the persistent instances of infections, the exacerbated hypoxia, the impaired nutrient conveyance, as well as the attenuated processes of angiogenesis and re‐epithelialization.

**Figure 2:**
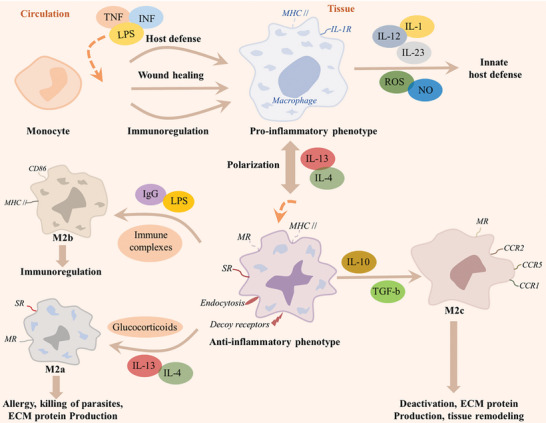
Schematic diagram of macrophages, derived from blood monocytes, play a critical role in wound healing, exhibiting pro‐inflammatory or pro‐reparative phenotypes. Polarization to these states is influenced by stimuli like glucocorticoids and LPS, leading to M2a, M2b, or M2c subtypes that modulate inflammation and facilitate tissue repair.

In the intricate milieu of chronic wound healing, the perpetual activation of both innate and adaptive immune responses is a hallmark. The innate immune system assumes a fundamental role in orchestrating the initial immune responses by discerning pathogenic signals and igniting a robust pro‐inflammatory cascade. Subsequently, the adaptive immune system operates diligently to eliminate damaged cells and pathogens from the wound site, imprinting a lasting immunological memory poised to elegantly combat potential future challenges in pathogen exposure.^[^ ^22^ ^]^ Specifically, macrophages play an integral role in diverse immune responses, including the establishment of adaptive immunity. They are indispensable in holding host immune functions across various physiological conditions, encompassing health, disease, wound healing, and regulation (**Figure** **2**).^[^ ^17^, ^23^ ^]^ Macrophages spearhead the initial inflammatory response, crucially clearing debris via phagocytosis, bolstering innate immunity, and propelling wound healing.^[^ ^24^ ^]^ In this epoch, the innate immune cascade is amplified by a fibroblast growth factor (FGF) and cytokines‐FGF, transforming growth factor‐beta (TGF‐β), vascular endothelial growth factor (VEGF)‐catalyzing cell activation and weaving the tapestry of tissue regeneration.^[^ ^25^ ^]^ Furthermore, as the reparative mechanisms commence, there is a notable transformation in the macrophagic phenotype from one that is pro‐inflammatory to one that is pro‐resolving. In their activated condition, the classically activated macrophages, which epitomize the pro‐inflammatory phenotype, are adept at recognizing molecular signatures associated with pathogenic entities, tissue trauma, and substances such as peptidoglycans, exemplified by the liberation of intracellular proteins and nucleic acids. These macrophages, characterized by their pro‐inflammatory functionality, are endowed with the capacity to metabolize antigens and to escalate the secretion of pro‐inflammatory cytokines, including tumor necrosis factor‐α (TNF‐α), interleukins IL‐1, interleukin‐6 (IL‐6), and IL‐12. Additionally, they are capable of generating oxidative metabolites, such as nitric oxide, in response to tissue distress.^[^ ^26^ ^]^ In concert with the host's immunological defense, macrophages endowed with a pro‐inflammatory profile are of significant importance in augmenting the organism's arsenal of defense mechanisms. These cells are not solely essential in the cathartic removal of tissues that have fallen victim to injury; they also exert a critical influence on the facilitation of phagocytosis and the genesis of neovascularization, processes that are indispensable for the restoration and reconstitution of tissue integrity. However, the protracted presence of such pro‐inflammatory macrophages within the systemic environment can precipitate a chronic inflammatory state, a condition that, if unattended, may culminate in adverse and potentially detrimental outcomes. Concurrently, the alternative activation of macrophages, indicative of an anti‐inflammatory phenotype, signals the onset of the resolution phase. These cells possess the unique ability to secrete an array of anti‐inflammatory cytokines that are instrumental in attenuating inflammatory responses and initiating the cascade of tissue repair. Among the cytokines secreted are interleukin IL‐4 and interleukin‐10 (IL‐10), which are renowned for their immunomodulatory capabilities; platelet‐derived growth factor (PDGF), which is fundamental to the process of tissue repair; TGF‐β, a pleiotropic cytokine that plays a multifaceted role in cellular proliferation and differentiation; VEGF, which is indispensable for angiogenesis; FGF, a potent catalyst for cell growth and tissue repair; and epidermal growth factor (EGF), which is crucial for the regeneration of epithelial tissues. In unison, these factors orchestrate the complex and nuanced sequence of events in the wound healing process, ensuring the restitution of tissue structure and functionality.^[^ ^25b^ ^]^ In the quest to restore dermal integrity, anti‐inflammatory macrophages play a seminal role in fostering the synthesis, reconstitution, and architectural restructuring of the ECM, as well as in the induction of angiogenesis, which are fundamental processes in the wound healing trajectory.^[^ ^27^ ^]^ In the harmonious regulation of cutaneous homeostasis, a synergistic interplay between regulatory T cells (T_regs_) and Th2 cells is observed, where they jointly facilitate the polarization of macrophages toward an anti‐inflammatory phenotype. This is mediated through the orchestrated secretion of a suite of cytokines, including IL‐10, Transforming Growth Factor‐beta 1 (TGF‐β1), IL‐4, IL‐5, IL‐13, and IL‐21, which are indispensable for the orchestrated formation of the ECM in a carefully controlled immunological cascade.^[^ ^28^ ^]^ Amidst the elaborate choreography of the wound‐healing process, innate immune cells are allocated distinctive roles, with their contributions being integral to the overall response. The engagement of adaptive immune cells, conversely, is nuanced and contingent upon the intricacy and magnitude of the injury sustained. In this context, LCs, a specialized subset of DCs, have emerged as key orchestrators in the facilitation and acceleration of the resolution phase of diabetic foot ulcers (DFUs). Their strategic involvement is notable, particularly within the complex milieu of DFU pathophysiology, where their multifaceted functions are indispensable for effective wound management and the restoration of tissue integrity.^[^ ^29^ ^]^

The intricate dynamics of chronic wound healing and its eventual resolution are significantly affected by a myriad of factors, including the patient's chronological age and the coexistence of other medical conditions.^[^ ^28b^ ^]^ Among diabetic individuals, the attenuated responsiveness of endothelial cells impedes the secretion of cytokines, culminating in a retardation of the angiogenesis process. Additionally, diminished oxygen saturation adversely affects the efficacy of immune cell function, thereby increasing the susceptibility to acute ulceration of wounds.^[^ ^30^ ^]^ In particular scenarios, wounds that heal at a languid pace are more prone to colonization by ambient bacteria, exemplified by Staphylococcus aureus (S. aureus), which poses a risk of inciting bacteremia and sepsis.^[^ ^18^ ^]^ Consequently, chronic wounds are identified as a predominant etiology for the necessity of limb amputations. It is thus imperative to attain an exhaustive comprehension of the immunological nuances inherent to chronic wounds to facilitate a more efficacious approach to disease stewardship.

Recognizing the parallels, autoimmune disorders, akin to chronic skin lesions, are characterized by a dysregulated immunological assault on the body's tissues and cells, resulting in substantial harm and the potential for organ dysfunction. These conditions are a leading cause of global morbidity and mortality, occupying the third position, and are precipitated by a sophisticated interplay between genetic susceptibilities and environmental provocations that disrupt immune homeostasis.^[^ ^31^ ^]^ Within the realm of autoimmunity, central tolerance is orchestrated by the vigilant activity of CD25fl Tregs, which are tasked with the eradication of errant T and B cells. Concurrently, these regulatory cells are instrumental in the synthesis of anti‐inflammatory cytokines and the curtailment of pro‐inflammatory cytokines, thereby facilitating the efficient clearance of apoptotic cells and maintaining immunological balance.^[^ ^32^ ^]^ Virus‐induced infections are often at the helm of inciting autoimmune diseases, as they induce subtle aberrations in antibody production. This perturbation can result in a misguided immune response, where B cells, intended to produce antibodies for foreign pathogens, instead target the individual's tissues. This pathogenic mechanism has been implicated in a spectrum of autoimmune afflictions, including but not limited to Type 1 diabetes mellitus, inflammatory bowel disease, and lupus erythematosus.^[^ ^33^ ^]^

## 3 Advanced Regenerative and Therapeutic Strategies of Hydrogel‐Based Strategies

The use of biocompatible materials that reduce allergens and immune responses has been adopted to promote wound healing. These materials facilitate regulated oxygen transfer, creating an atmosphere conducive to the healing of persistent wounds.^[^ ^34^ ^]^ In this emerging field, innovative materials such as hydrocolloids, hydrogels and other bio‐functional polymers are being investigated for their potential in chronic wound management. These materials demonstrate exceptional biocompatibility and a unique ability to encapsulate a variety of cellular components and biomacromolecules, positioning them as leading contenders in wound care. Their controlled release capabilities under various external stimuli make them particularly promising for modulating and maintaining the physiological microenvironment of the wound, thereby enhancing the healing process.^[^ ^35^ ^]^ Among them, hydrogels emerge as water‐swollen 3D polymer networks that efficiently absorb biological fluids. They possess customizable physicochemical properties and serve as a flexible foundation for a range of medical applications. Through computational design schemes, materials science is used to tailor their properties and control cellular communication processes and fates.^[^ ^36^ ^]^ Hydrogels, with their ECM‐like properties, malleability and range of biological functions, are ideally suited for the precise delivery of immunomodulatory substances and cellular elements.^[^ ^37^ ^]^ They provide a favorable microenvironment for the attraction, activation and proliferation of immune cells, facilitating local immune regulation. Although their high‐water content and reduced mechanical resilience limit exudate absorption, hydrogels maintain a hydrated wound environment which is beneficial to the healing process. Their pliable and elastic consistency allows for effortless application and painless removal, coupled with a soothing cooling sensation that reduces the heat and discomfort associated with skin injuries, making them beneficial for wound management.^[^ ^38^ ^]^

Due to their innate ability to mimic the ECM and their tunable physical and chemical properties, hydrogels are lauded for their ability to encapsulate and proliferate cells both in vitro and in vivo, acting as a potent catalyst for efficient tissue regeneration. For example, a range of cells, including stem cells, islet cells, hepatocytes and endothelial cells (ECs), are able to proliferate while retaining their functional properties in vitro when encapsulated in hydrogels. Among these, stem cells play a pivotal role in regenerative medicine, particularly in the treatment of chronic inflammatory and autoimmune diseases, thanks to their distinctive pluripotency and potent immunomodulatory functions, driving their exploration in clinical trials. Their efficacy is particularly evident in the secretion of bioactive factors that suppress the proliferation of immune cells and the secretion of pro‐inflammatory cytokines, thus helping to alleviate inflammatory responses. In addition, stem cells have the ability to engage in a direct dialogue with immune cells, subtly influencing their activation and functionality. Such interactions are crucial as they promote a state of immune tolerance and are essential for maintaining tissue homeostasis.^[^ ^20^ ^]^ They were then applied to specific sites to act as a hub for the production of proteins and factors, thereby continuously facilitating and triggering the healing of chronic wounds. Like nanocarriers in drug delivery, hydrogels are considered perfect candidates for the controlled and sustained release of therapeutics at specific sites to assess the efficacy of wound healing. As a result, the convergence of these advanced material‐based treatments is poised to transform wound care, improve outcomes and reduce healthcare costs.

Chronic wounds are a major clinical challenge, causing ongoing discomfort and a significant decline in patients’ quality of life.^[^ ^39^ ^]^ The persistent exudate and free radicals in these wounds create an environment conducive to microbial growth and inflammation, inhibiting the natural healing process. Innovative therapeutic strategies are emerging that include skin substitutes, cellular treatments and advanced biomaterials for wound care, including hydrogels and adaptive dressings.^[^ ^18^ ^]^ Here, we will examine the function of hydrogels in the management of chronic wounds and present the amalgamation of various hydrogel‐derived nanocarriers with cell therapy, focusing on the collaborative impact of these hydrogel‐based nanocarriers in improving therapeutic outcomes.

### 3.1 Hydrogel Innovations in Chronic Wound Management

There have been significant advances in the management of chronic wounds, particularly DFUs, with autologous skin grafting being a cornerstone of treatment.^[^ ^40^ ^]^ This involves the careful development of stratified epidermal cultures using autologous fibroblasts derived from 3 mm punch biopsies, which are then applied to a dermal scaffold. In cases where autograft sources are scarce, such as after major trauma or severe burns, cadaveric dermal allografts have emerged as a viable therapeutic alternative for a range of wounds and injuries.^[^ ^41^ ^]^ Yet, the scarcity of autograft sources, which is exacerbated in cases of major trauma or chronic wounds, presents a critical challenge, particularly after severe burns. Within this framework, cadaveric dermal allografts have emerged as a viable therapeutic contender, applicable to a range of wounds and injuries.^[^ ^42^ ^]^

**Figure 3:**
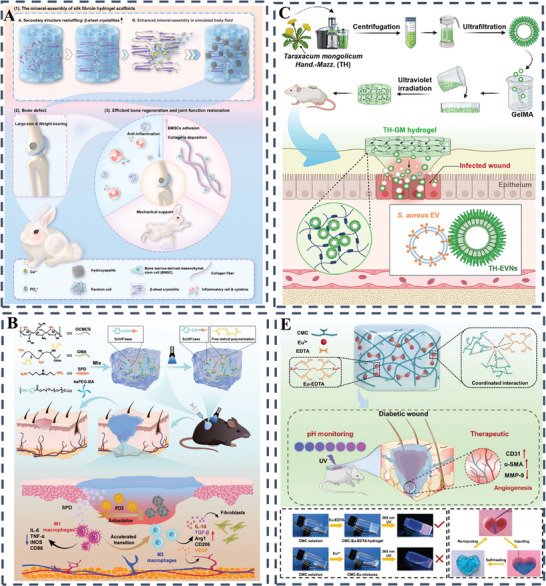
A) A diagrammatic representation of the assembly process for a mineralized silk fibroin hydrogel scaffold, featuring a biomimetic 3D architecture, appropriate mechanical characteristics, and superior osteoinductive qualities to facilitate swift bone healing. Reproduced with permission.^[^ ^43^ ^]^ Copyright 2024, KeAi Communications Co; B) Schematic depiction of a photoactivated hydrogel integrated with dandelion‐derived extracellular vesicle‐like nanoparticles, designed for the treatment of wounds inflicted by Staphylococcus aureus exotoxins.^[^ ^44^ ^]^ Copyright 2024, Elsevier; C) A visual depiction of the fabrication procedure for the DN‐SPD hydrogel and its capacity to regulate immune responses for effective wound management.^[^ ^45^ ^]^ Copyright 2024, Elsevier; D) A schematic depiction of the CMC‐Eu‐EDTA hydrogel's development and its role in promoting the recovery of diabetic wounds.^[^ ^46^ ^]^ Copyright 2024, Wiley‐VCH Verlag.

For example, Huang et al. developed a silk fibroin‐based hydrogel scaffold using an “organic‐inorganic assembly” strategy to enhance its osteogenic properties for rapid bone regeneration. The scaffold, fabricated by digital light processing 3D printing, demonstrated biomimetic mechanical properties and promoted efficient bone healing in weight‐bearing defects within one month. This approach represents a promising alternative to traditional bone grafts, providing a cell‐free, growth factor‐free solution for large and weight‐bearing bone regeneration (**Figure** **3**A).^[^ ^43^ ^]^ Continuing this innovative trajectory in biomaterials for regenerative medicine, another study has presented a novel wound dressing. This dressing, which incorporates dandelion‐derived extracellular vesicle‐like nanoparticles (TH‐EVNs) embedded in a gelatin methacryloyl (GelMA) hydrogel, demonstrates the unique ability of TH‐EVNs to neutralize Staphylococcus aureus exotoxins, a critical factor in the management of invasive wounds. The hydrogel dressings have demonstrated remarkable detoxification properties, accelerated re‐epithelialization, enhanced collagen maturation and significant anti‐inflammatory effects upon in vivo evaluation, representing a promising therapeutic modality for wounds caused by S. aureus exotoxins (Figure 3B).^[^ ^44^ ^]^ Transitioning from bone to soft tissue repair, the field has witnessed the development of a spermidine‐functionalized, injectable hydrogel that significantly accelerates the healing of both acute and diabetic wounds by reducing inflammation and enhancing tissue regeneration. This double‐network hydrogel, which can be photo‐crosslinked in situ, exhibits excellent mechanical properties and biocompatibility, and its immunomodulatory effects promote a shift in macrophage polarization toward a regenerative phenotype. The study demonstrates the potential of locally administered spermidine in biomaterials to mitigate foreign body reactions and offers a promising approach for treating chronic wounds (Figure 3C).^[^ ^45^ ^]^ In the pursuit of more sophisticated wound care solutions, another study presents the development of a self‐healing, injectable hydrogel dressing designed for monitoring and therapy of diabetic wounds. The hydrogel, prepared by cross‐linking carboxymethyl cellulose with europium‐ethylenediaminetetraacetic acid (Eu‐EDTA) complexes, not only demonstrates enhanced angiogenesis and real‐time pH monitoring capabilities but also actively promotes wound healing by upregulating angiogenesis‐related factors and downregulating matrix metalloproteinase‐9 (MMP‐9). This innovative hydrogel shows significant potential for clinical applications in diabetic wound management, marking another stride in the evolution of hydrogel‐based therapies (Figure 3D).^[^ ^46^ ^]^ However, despite the arsenal of immunosuppressive strategies in allogeneic transplants, the threat of premature rejection continues to be a daunting barrier to the universal adoption of allografts.

### 3.2 Advanced Chronic Wound Care by Integrating Hydrogel‐Based Nanomaterials with Cell Therapy

Hydrogel nanocarriers are emerging as a promising platform due to their biocompatibility and tunable physical properties, which create a microenvironment conducive to supporting cell survival, proliferation and differentiation.^[^ ^20^ ^]^ Innovative biomaterials materials such as hydrogels, known for their biocompatibility and ability to encapsulate a variety of cells and bio‐macromolecules, are being tailored to modulate immune cell polarization and provide a conducive microenvironment for chronic wound healing.^[^ ^47^ ^]^ The composition of these hydrogels allows the encapsulation of key molecules and cells. Significantly, cell‐based therapies have shown variable success in clinical trials due to challenges such as low survival rates and uncontrolled cell differentiation. However, the integration of cell therapy with nanocarriers, particularly hydrogel‐based ones, has shown significant potential for improving tissue regeneration. Consider Integra, a collagen‐ and glycosaminoglycan‐enriched, silicone‐coated 3D dermal matrix that has accelerated the healing process in clinical trials. It has also been shown to be highly effective in treating severe wounds, particularly in military personnel, with a success rate of 78%–86%. What sets Integra apart is its ability to promote substantial dermal reconstruction, characterized by improved durability and strength, which has been instrumental in its track record in managing complex wounds and aiding patient rehabilitation.^[^ ^48^ ^]^ Hyalomatrix, a commercially available biodegradable dermal matrix with an esterified hyaluronic acid (HA) contact layer known as Hyaff (Medline), enhances cellular activity and ECM assembly when applied to deep wounds.^[^ ^49^ ^]^ Together, Integra and Hyalomatrix form a complementary duo that pushes the boundaries of tissue engineering and provides a holistic approach to the treatment of complex wound scenarios.

**Figure 4:**
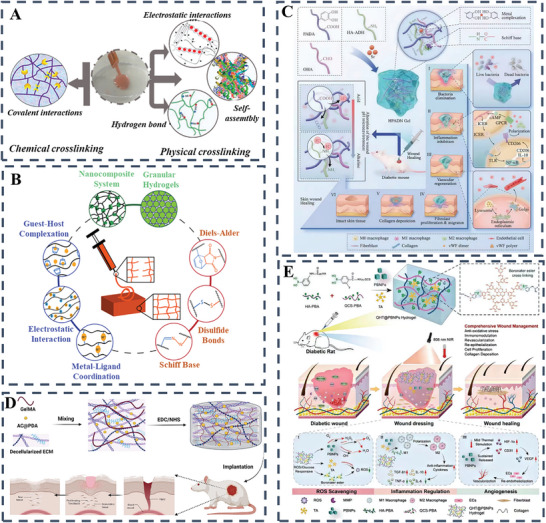
A) Utilizing a combination of chemical and physical bonding to fabricate diverse hydrogel types for a wide range of uses. Reproduced with permission.^[^ ^1^ ^]^ Copyright 2021, Wiley‐VCH; B) Polymer reversibility dictates hydrogel's thinning and self‐healing dynamics. Reproduced with permission.^[^ ^50^ ^]^ Copyright 2020, Wiley; C) Schematic illustration of the preparation, pH regulation mechanism, and skin repair mechanism of the HPADN hydrogel.^[^ ^51^ ^]^ Copyright 2023, Wiley‐VCH Verlag; D) Bioactive hydrogels from decellularized ECM, GelMA, PDA‐AC nano for skin regeneration. Reproduced with permission.^[^ ^52^ ^]^ Copyright 2023, Elsevier; E) Enhanced tissue‐integrated GelMA‐AlgMA hydrogels through covalent and Ca^2+^ dual crosslinking. Reproduced with permission.^[^ ^53^ ^]^ Copyright 2020, Wiley.

Currently, a variety of polymers, both natural and synthetic, are used in conjunction with different cross‐linking methods to produce hydrogels. The cohesion of these aqueous gels is primarily determined by the chemical and physical bonds that exist between segments along the polymer backbone. These bonds are critical in determining the rate at which the hydrogels degrade, and their overall structural integrity once introduced into the body (**Figure** **4**A). For example, covalent crosslinking is often used to develop 3D hydrogel networks suitable for efficient encapsulation of cells and biomolecules such as proteins and growth factors.^[^ ^1^ ^]^ Injectable hydrogels have gained significant utility in biomedicine, particularly for the targeted delivery of various bioactive components.

For example, Zhao et al. developed a GelMA hydrogel integrated with human umbilical vein endothelial cells (HUVECs) to facilitate full‐thickness skin wound healing. This GelMA hydrogel not only effectively addressed wound healing, but also ensured prolonged exosome release.^[^ ^50^ ^]^ In this scenario, the fusion of chemically cross‐linked hydrogels with additional binding mechanisms such as hydrogen and electrostatic forces led to the creation of interpenetrating polymer networks (IPNs) with reversible linkages and significant mechanical properties (Figure 4B).^[^ ^50^ ^]^ Similarly, Xia et al. have developed a glycopeptide‐based hydrogel to modulate the pH microenvironment of diabetic wounds to promote healing. The hydrogel, composed of modified HA and poly(6‐aminocaproic acid), forms a network that reduces inflammation and promotes angiogenesis. It promotes the polarization of M2 macrophages, which is crucial for healing, and represents a promising strategy for the treatment of diabetic wounds (Figure 4C).^[^ ^51^ ^]^ Researchers are continually advancing the field of hydrogel development for medical applications, and Professor Fan has made a significant contribution by developing a bioactive hydrogel from decellularized ECM, GelMA and AC@PDA nanoparticles that promotes wound healing with improved elasticity and shape recovery, despite a slight reduction in compressive strength; This hydrogel excels in moisture retention and mechanical performance, forming an interpenetrating network with EDC/NHS, and SEM analysis confirms its excellent water absorption and wound closure in mice, indicating the potential for scarless healing, positioning it as a leading next‐generation wound dressing (Figure 4D).^[^ ^52^ ^]^ Besides, severe oxidative stress, inflammation, and poor angiogenesis often impede the healing of diabetic wounds. A new class of bioactive hydrogels, featuring immunomodulation and angiogenic properties, has been engineered to facilitate healing without additional drugs. Guo et al. developed a novel hydrogel, cross‐linked by dynamic borate bonds, integrates phenylboronic acid‐modified chitosan, HA, and Prussian blue nanoparticles (PBNPs) to modulate the wound environment. The obtained QHT@PBNPs exhibited broad‐spectrum antioxidative activity and transforms the inflammatory milieu through HA's macrophage regulation. The mild photothermal effect of PBNPs further boosts angiogenesis, epithelialization, and collagen deposition, offering a non‐pharmacological approach to diabetic wound care (Figure 4E).^[^ ^53^ ^]^

In the absence of key active ingredients, many hydrogel systems have failed to accelerate chronic wound healing. However, the integration of various bioactive components can significantly promote the healing process. Based on their level of bioactivity, hydrogel‐based wound dressings can be classified into three distinct groups: passive, active and responsive.^[^ ^54^ ^]^ For example, Gauze interacts passively with the wound, while soft non‐woven pads, as bioactive dressings, absorb exudate and keep the wound moist, reducing moisture‐induced skin damage.^[^ ^55^ ^]^ Many wound care formulations have been developed using alginate and collagen as active dressings for dermatological healing.^[^ ^56^ ^]^ For example, alginate‐derived hydrogels are highly absorbent and contribute to hemostasis, while collagen‐derived hydrogels promote collagen synthesis, which is essential for the wound healing process.^[^ ^57^ ^]^ Hydrogel‐based materials have been used as responsive wound dressings, capable of adapting to the conditions of the wound microenvironment and engaging with skin tissues to enhance the healing process. For example, GAG‐derived hydrogels such as HA are recognized for their interactive properties in dressings that facilitate wound healing.^[^ ^58^ ^]^

**Figure 5:**
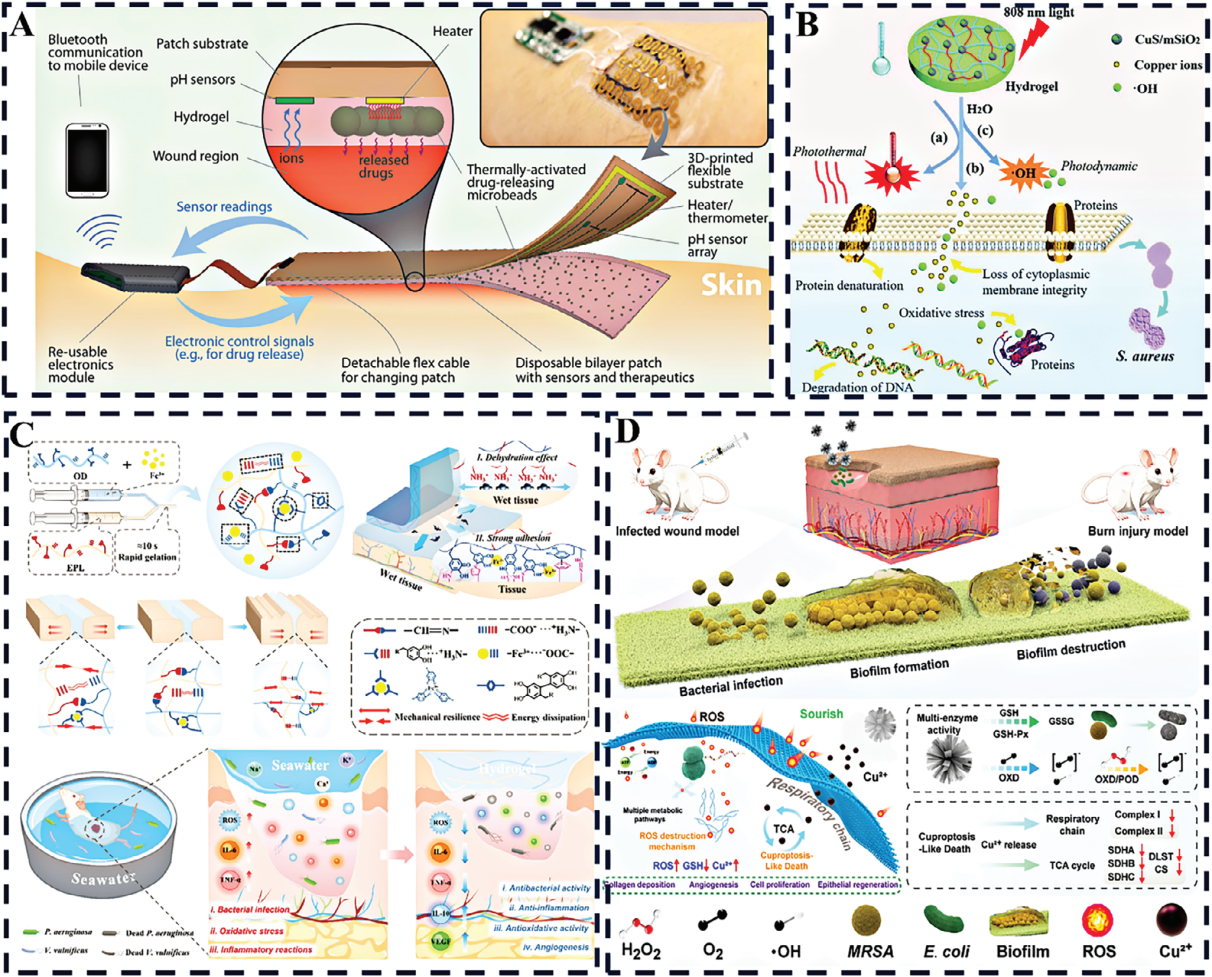
A) Smart bandage integrates flexible pH sensors and a heater with thermo‐responsive drug carriers in alginate hydrogel, connected wirelessly to an electronic module for monitoring and control. Reproduced with permission.^[^ ^59^ ^]^ Copyright 2018, Wiley‐VCH; B) NIR‐activated hybrid hydrogel eradicates bacteria via CuS nanoparticle ion release and hydroxyl radical generation. Reproduced with permission.^[^ ^61^ ^]^ Copyright 2018, Royal Society of Chemistry; C) Multi‐crosslinked hydrogels offer mechanical resilience and energy dissipation, designed to adapt to dynamic environments with robust structural integrity. Reproduced with permission.^[^ ^62^ ^]^ Copyright 2023, Elsevier; D) Synthesis of CuCo_2_O_4_; biofilm lifecycle and degradation; multi‐enzyme synergy and cuproptosis‐induced antibacterial effects.^[^ ^63^ ^]^ Copyright 2024, ACS.

Interactive and bioactive hydrogels can be designed by integrating a range of active ingredients to provide adaptive functionality throughout the wound healing process. However, their performance is often limited by insufficient gas and oxygen permeability. To address this, hydrogels are enhanced with antimicrobial agents, such as antimicrobial peptides (AMPs) and antioxidants, which enhance their bactericidal properties and overcome permeability limitations, thereby increasing their protection against infection.^[^ ^6^, ^34^ ^]^ For instance, Mostafalu's team has introduced an innovative smart hydrogel, integrating temperature and pH sensors into its flexible fabric to address a significant medical need by providing continuous wound health monitoring (**Figure** **5**A).^[^ ^59^ ^]^ Addressing the challenges of antimicrobial resistance and the hazards of direct application of bioactive agents, a novel integrated hydrogel‐bioactive system represents a significant therapeutic advance that competently manages contaminated wounds and mitigates potential adverse effects. The hydrogels are formulated with a wide range of bioactive agents and incorporate a variety of materials such as specialized ceramics, metallic elements, various polymers, advanced nanocomposites and carbon‐derived materials. In addition, they are infused with natural components known for their therapeutic benefits, such as the antimicrobial and wound‐healing properties of honey, as well as potent bioactive peptides.^[^ ^6^, ^45^ ^]^ They have also been used to stimulate cell proliferation and migration during the maturation phase of the ECM in wound healing. In addition, hybrid hydrogels infused with bioactive agents can modulate macrophage behavior to promote wound healing and tissue regeneration.^[^ ^60^ ^]^ Bacterial infections often impede the healing process of injured tissues, necessitating the enhancement of local antibacterial capabilities. Therefore, Wu et al. developed a hybrid hydrogel synthesized by radical polymerization and integrated with 3‐(trimethoxysilyl)propyl methacrylate and mesoporous silica‐modified CuS nanoparticles to significantly enhance antibacterial activity and wound healing. This hydrogel, which kills over 99% of Staphylococcus aureus and Escherichia coli within 10 min under 808 nm NIR light, does so through hyperthermia and the emission of reactive oxygen species (ROS) and copper ions. The hydrogel, composed of N‐isopropylacrylamide and acrylamide, also stimulates fibroblast proliferation and angiogenesis, thereby accelerating skin regeneration (Figure 5B).^[^ ^61^ ^]^ Wounds in seawater face problems such as tissue damage and infection due to the salty environment and bacteria, and current hydrogels don't adhere well or fight bacteria effectively in these conditions. Consequently, the OD/EPL@Fe hydrogel, a blend of catechol‐modified HA, ε‐poly‐L‐lysine and Fe^3+^, provides enhanced wet adhesion of 78 kPa, surpassing conventional adhesives. It fights marine bacteria, inhibits biofilm and is removable with deferoxamine mesylate. This injectable, self‐healing hydrogel scavenges ROS, provides photothermal action and promotes hemostasis, effectively isolating wounds from seawater. The hydrogel binds effectively to wounds in rat models, providing protection against infection and promoting rapid skin healing in seawater‐exposed wounds (Figure 5C).^[^ ^62^ ^]^ Nanozyme therapy shows potential for biofilm wounds but is limited by material efficiency. In a significant advance in nanozyme therapy, Wang et al. have developed CuCo_2_O_4_ nanoflowers that exhibit potent antibacterial activity against MRSA‐infected wounds. These nanozymes, engineered with dual peroxidase and oxidase activities, catalyze the generation of ROS that effectively disrupt bacterial biofilms and accelerate the healing process. The unique structure of the nanoflowers, achieved through hydrothermal synthesis, not only enhances their catalytic efficiency, but also promotes tissue regeneration in vivo, overcoming the challenges associated with biofilm‐induced impaired healing (Figure 5D).^[^ ^63^ ^]^

**Figure 6:**
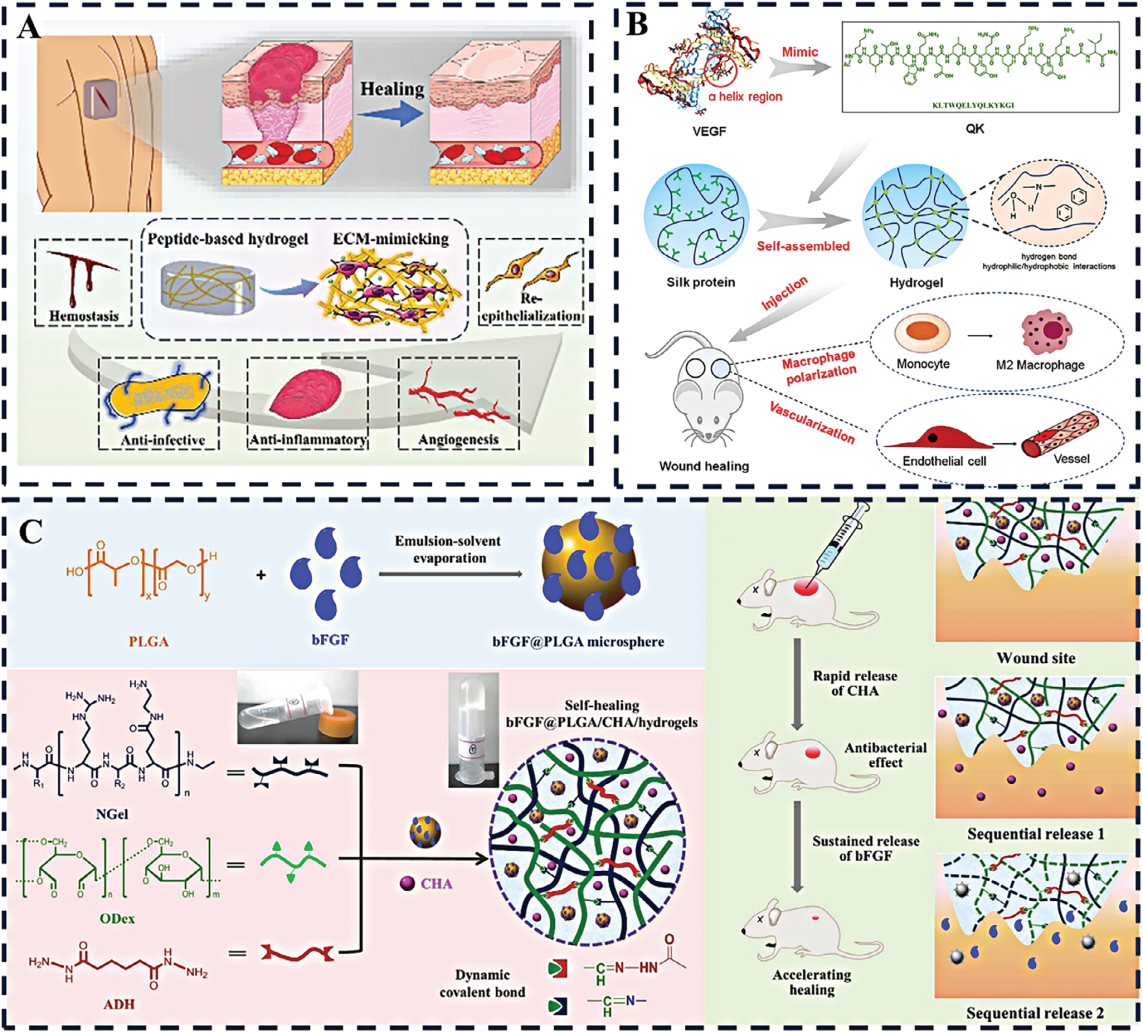
A) Schematic representation depicting the impact of peptide‐based self‐assembling hydrogels on wound healing dynamics. Reproduced with permission.^[^ ^64^ ^]^ B) Schematic diagrams depicting the fabrication of QK‐SF hydrogel and its wound healing mechanism. Reproduced with permission.^[^ ^66^ ^]^ Copyright 2023, Wiley‐VCH Verlag; C) Diagrammatic depiction of bFGF@PLGA/CHA‐enriched hydrogels designed for self‐repair and staged drug release, aimed at curbing wound infections and enhancing the healing process. Reproduced with permission.^[^ ^67^ ^]^ Copyright 2019, Elsevier.

A range of synthetic and natural peptides have been masterfully incorporated into hydrogel formulations as key bioactive agents. These specific peptide sequences are capable of self‐assembling into intricate molecular structures under the influence of subtle internal or external stimuli. The cleverly designed self‐assembling peptides offer a wide range of applications, such as mimicking the ECM, stimulating the immune response and facilitating targeted drug delivery (**Figure** **6**A).^[^ ^64^ ^]^ Advances in peptide‐based wound care are driving the development of specialized materials to address specific healing needs. In this work, silk fibroin‐based nanosheets, known for their high transparency and flexibility, were loaded with an integrin‐binding peptide to enhance cell survival. This advanced material induced the expression of angiogenic markers (including VEGF and CD31), facilitating rapid healing of DFUs.^[^ ^65^ ^]^ People with diabetes who struggle with DFUs face higher rates of disability, affecting 15–20% and causing significant personal and societal economic burden. Therefore, Chen et al. have developed a QK‐SF hydrogel that combines silk fibroin with a VEGF mimetic peptide for enhanced tissue regeneration. This dual‐functional bioactive gel promotes vascularization and macrophage polarization, which are essential for healing. With excellent stability and low cytotoxicity, it induces M2 macrophage polarization and stimulates angiogenesis in HUVECs. In vivo, it accelerates wound healing by promoting M2 phenotype, collagen deposition and reducing inflammation, demonstrating its promise as an advanced wound dressing for tissue repair and regeneration (Figure 6B).^[^ ^66^ ^]^ Building on this foundation, the quest for innovation in wound care continues, addressing the specific shortcomings of existing treatments. Similarly, in response to the limitations of conventional injectable hydrogels in wound healing, a novel hydrogel with dynamic covalent self‐healing, antibacterial properties and sequential drug release capabilities has been developed. This injectable and self‐healing hydrogel, synthesized by the dynamic reaction of aminated gelatin, adipic acid dihydrazide and oxidized dextran, exhibits remarkable self‐healing ability via dynamic imine and acyl hydrazone bonds. By incorporating bFGF@PLGA microspheres and chlorhexidine acetate, this hydrogel shows excellent biodegradability and morphological stability after swelling, allowing the sequential release of CHA and the peptide growth factor bFGF. Application of these hydrogels to rat wound skin effectively prevents infection through rapid release of CHA and promotes cell proliferation and wound healing through sustained release of bFGF. This innovative self‐healing hydrogel with sequential drug release properties showed great promise as an advanced wound hydrogel (Figure 6C).^[^ ^67^ ^]^

**Figure 7:**
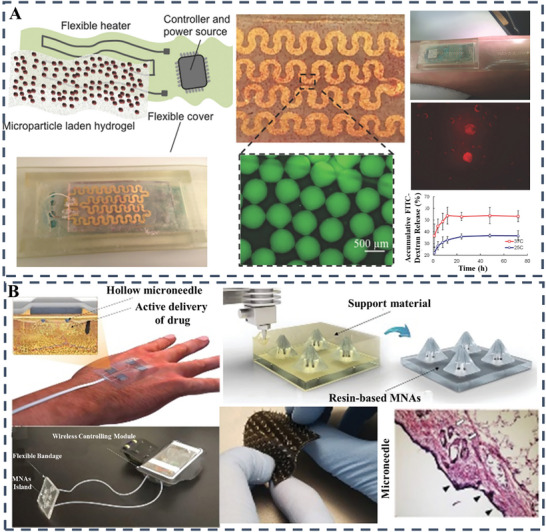
A) Integrated wound dressing featuring drug‐loaded microcarriers and electronics includes detailed schematic imagery and introduces a wireless smart bandage system. Reproduced with permission.^[^ ^73^ ^]^ Copyright 2016, Wiley; B) Dual‐module drug delivery bandage is a wearable system with integrated microneedle arrays and features wireless control via smartphone for adjusting the drug release rate. Reproduced with permission.^[^ ^74^ ^]^ Copyright 2020, Wiley‐VCH Verlag.

Wound healing materials now incorporate complex delivery systems designed to harness a range of growth factors and cytokines, focusing on their essential roles at different stages of the healing process.^[^ ^68^ ^]^ The use of these platforms results in a reduced total drug payload, thereby reducing distal effects and associated side effects compared to infusion.^[^ ^69^ ^]^ FGF and EGF, essential for epithelial regeneration, are the mainstays of wound repair, while the synergy of pro‐angiogenic factors such as Ang2 and VEGF promotes angiogenesis by facilitating the release of pericytes and the formation of vascular networks.^[^ ^70^ ^]^ Improving the stability and maintaining the efficacy of growth factors necessitates meticulous molecular design and the use of sophisticated delivery systems, as extensively documented in scholarly works.^[^ ^71^ ^]^ However, the practical application of these methodologies in clinical settings encounters challenges due to the rapid depletion of bioactive compounds from the matrices and the induction of undesirable foreign body responses upon additional molecule integration. To overcome this obstacle, a promising strategy involves the precise delivery of drug molecules to the epidermal layer of the skin. Within this framework, advanced intelligent hydrogels, which are sensitive to various triggers such as temperature changes, pH variations and glucose concentrations, have been ingeniously designed to intricately control the release dynamics of bioactive agents.^[^ ^72^ ^]^ For instance, in field of skin health and wound care, precise topical delivery of medications and growth factors via an innovative dermal patch with thermos‐responsive drug microcarriers and integrated flexible heater technology shows great promise for enhancing healing. This creative patch conforms closely to the wound area, enabling controlled drug release through electronic temperature modulation of the hydrogel layer. Utilizing microfluidic fabrication, uniform thermos‐responsive microcarriers encapsulating bioactive molecules ensure precise regulation of release kinetics. This advancement signifies a significant leap toward sophisticated, self‐regulating systems for topical skin therapy and wound (**Figure** **7**A).^[^ ^73^ ^]^ In a different research investigation, the goal is to ensure sufficient transportation of bioactive substances to address exuding long wounds. Specifically, a progressive programmable platform has been devised for the active delivery of a diverse array of drugs with tailored temporal profiles using microscale needles that penetrate deep into the wound bed layers. The targeted administration of VEGF through these microscale needle arrays underscores the significant role of not only selecting suitable therapeutics but also optimizing delivery methodology and spatial distribution within the wound bed. Using this advanced system to deliver VEGF to persistent skin wounds in diabetic rodents results in significant improvements in wound healing, tissue regeneration, blood vessel formation and follicular recovery compared to standard topical treatments (Figure 7B).^[^ ^74^ ^]^ In a fascinating research endeavor, a specialized glucose‐responsive complex was developed to facilitate the controlled release of insulin from tert‐butyl (2‐acrylamidoethyl) carbamate (Boc‐EDAA). When exposed to hyperglycemic conditions, the glucose‐sensitive phenylboronic acid (PBA) could alter the polymer's surface charge, transitioning it from positive to negative and thereby enabling the targeted release of insulin. This innovative approach was further validated through in vivo experiments, highlighting the swift and precise responsiveness of the advanced hydrogel system, ultimately resulting in the liberation of insulin triggered by elevated glucose levels.^[^ ^72^ ^]^

One prevalent approach in wound healing involves regulating immune responses by releasing cytokines, inhibitors of proteases, growth factors, microRNAs (miRNAs), and small interfering RNAs (siRNAs). The significant inflammation seen in chronic wounds and scar formation has underscored the importance of monitoring immune responses to improve treatment outcomes. Therefore, a thorough examination of diverse methods for immune modulation is crucial for addressing the intricacies of managing chronic wounds and inhibiting formation.

## 4 Hydrogel Innovations in Immune Regulation: Addressing Chronic Skin Wounds

### 4.1 The Emergence of Hydrogel‐Based Strategies in Immunomodulation

Diverse signals from the cellular microenvironment, including biochemical cues from cell‐ECM interfaces, biophysical stimuli from external mechanical and electrical forces, and various signals mediated by biomaterials at cell‐material interfaces, influence cellular behaviors.^[^ ^75^ ^]^ The cumulative effect of these signals is vital for managing the equilibrium between inflammatory and anti‐inflammatory cell responses, thus promoting tissue repair. Selecting the right immunomodulatory hydrogel is key to attaining this equilibrium.^[^ ^20^ ^]^ Numerous hydrogel materials have been developed for immune modulation, with some triggering inflammatory responses and others lacking targeted immunomodulatory cues.

Aspects such as the level of cross‐linking density, the rate of degradation under physiological conditions, mechanical properties, hydrophilicity, surface chemistry, energy, topographical features, dimensions, shape and the specific formulation of the hydrogel are essential variables to be considered. The physicochemical properties of hydrogels play a significant role in modulating the intensity and characteristics of immune responses. Certain biomaterials have molecular signatures that resemble danger signals and pathogen markers. For example, the repetitive sequences within hydrogel polymers can induce immune responses similar to those induced by bacterial glycopolysaccharide layers. Additionally, specific types of micro and nanoparticles may mimic the dimensions of bacterial and viral.^[^ ^20^ ^]^ In a recent study, DCs were cultured on different bio‐derived hydrogels to assess the expression levels of maturation markers such as CD40. It was observed that hydrogels composed of alginate and agarose did not induce DC maturation; however, the application of chitosan hydrogels promoted the maturation of these cells. In addition, DCs cultured on chitosan‐derived hydrogels showed increased levels of pro‐inflammatory markers and increased production of pro‐inflammatory cytokines.^[^ ^76^ ^]^

In a separate study by Cha et al., it was found that integrins predominantly dictate the interactions between biomaterials and macrophages. Macrophages, derived from a variety of embryonic sources, are critical for tissue repair. They are activated by DAMPs upon injury, migrate to wound sites and secrete MCP‐1 to attract other macrophages. They also produce LTB4, which amplifies the inflammatory response and initiates the repair process.^[^ ^77^ ^]^ Once at the site of injury, macrophages aggressively remove debris and signal fibroblasts to regulate and repair the ECM.^[^ ^78^ ^]^ During the early stages of wound healing, macrophages adopt a pro‐inflammatory M1 phenotype, activated by DAMPs and inflammatory factors such as IFNγ and TNF. These M1 macrophages, known for their cytotoxic effects, are major producers of TNF and the cytokines IL‐1β and IL‐6.^[^ ^79^ ^]^ Later in wound healing, macrophages switch to the M2 phenotype triggered by IL‐4 and IL‐13, key cytokines in Th2 responses.^[^ ^80^ ^]^ This transition is also characterized by changes in macrophage metabolism.^[^ ^81^ ^]^ Macrophages switch to pro‐resolving by engulfing aged neutrophils, which triggers the production of inflammation‐resolving lipoxins.^[^ ^82^ ^]^ These compounds significantly reduce inflammation and promote tissue healing and balance.^[^ ^83^ ^]^ Pro‐resolving actions include the clearance of neutrophils that have undergone apoptosis, as well as the termination of inflammatory signaling pathways and the synthesis of eicosanoids.^[^ ^84^ ^]^ Lipoxins aid resolution by blocking neutrophil influx and directing monocyte clearance in the lymph nodes.^[^ ^85^ ^]^ In the vicinity of wounds, macrophages are thought to control several cell types – from fibroblasts that build tissue scaffolding, to endothelial cells that line blood vessels, to the less studied adipocytes and melanocytes – that play an important role in repair. Their regulatory role evolves with healing. Early macrophage depletion with diphtheria toxin can reduce new blood vessel formation, delay skin regeneration and reduce scarring, while later removal can cause bleeding and delay wound closure.^[^ ^86^ ^]^ Macrophages exhibited an inability to recognize cell attachment motifs on the biomaterial, leading to the production of pro‐inflammatory markers. Conversely, hydrogels containing specific attachment sites were found to support the expression of an anti‐inflammatory.^[^ ^87^ ^]^ Utilizing high molecular weight polymers like HA and chitosan in the creation of naturally derived hydrogels with ROS‐scavenging properties may serve as a viable alternative to the components missing in the ECM. These hydrogels have been shown to exhibit inherent anti‐inflammatory characteristics, potentially mimicking the ECM and providing benefits.^[^ ^88^ ^]^ The structural and biochemical diversity of HA macromolecules has a significant impact on immune cell responses. Immunomodulation is observed with high‐molecular‐weight HA, whereas low‐molecular‐weight HA tends to promote inflammatory reactions. Despite the similarity in their repeating disaccharide units, only low‐molecular‐weight HA interacts with Toll‐like receptors, triggering pro‐inflammatory effects through the engagement of these pattern receptors. Low‐molecular‐weight HA functions as a danger‐associated molecular pattern that stimulates the maturation of dendritic cells and promotes the release of pro‐inflammatory cytokines like TNF‐α, IL‐6, and IL‐12 by various cell types. It also enhances chemokine expression, cell trafficking, and proliferation. In contrast, high‐molecular‐weight HA inhibits inflammation and decreases the production of inflammatory cytokines by interacting with CD44, the primary HA‐binding transmembrane glycoprotein on cell surfaces.^[^ ^89^ ^]^ CD44 plays a crucial role in translating signals from the ECM, influencing cellular processes such as growth, activation, differentiation, and immune homeostasis, including the maintenance of Th1 memory cells. Additionally, the crosslinking of HA significantly modulates leukocyte function through interactions with hyaladherins, which are HA‐binding.^[^ ^90^ ^]^

High‐molecular weight HA's role in wound healing has spurred its widespread application in crafting immunomodulatory hydrogels for chronic wound treatment, as highlighted by Zamboni et al.^[^ ^90^ ^]^ Additionally, Wang et al. introduced a photoresponsive nanocomposite hydrogel based on HA to enhance dynamic macrophage immunomodulation. In their study, they linked photodegradation alkoxyl phenacyl‐based polycarbonate (APP) to an acrylate HA macromer (HA‐AC) to enable controlled release of the Arg‐Gly‐Asp (RGD) adhesive peptide. The incorporation of the RGD peptide could activate the αvβ3 integrin in macrophages, promoting a shift toward anti‐inflammatory macrophages.^[^ ^91^ ^]^

Furthermore, the impact of collagen‐based hydrogels on immune cells has been profound. Notably, the interaction between macrophage scavenger receptors and exposed collagen ligands has been shown to significantly enhance specific conformational outcomes. Moreover, these hydrogels have demonstrated a remarkable ability to mitigate the immunogenic response elicited by integrated allogeneic MSCs. Such findings exemplify the innovative potential of collagen‐based gels in shaping immune cell behavior and modulating the immunogenicity of co‐cultured cells.^[^ ^92^ ^]^ As an illustration, gelatin, a denatured form of collagen, was employed to regulate cellular reactions. In a particular investigation, monocytes were cultured on hydrogels made of GelMA.^[^ ^93^ ^]^ Demonstration of TNF‐α absorption by CD14+ monocytes on GelMA sparked the hypothesis that sequestration within GelMA diminishes soluble TNF‐α availability, thereby suppressing TNF‐α gene expression. Moreover, elevated expression of anti‐inflammatory cytokines IL‐10 and IL‐1RA, coupled with decreased levels of inflammatory markers iNOS and TNF‐α, was observed in macrophages cultured on GelMA versus PEGDA hydrogels. Notably, the significant role of integrin α2β1 in macrophage polarization was underscored, particularly in the promotion of an anti‐inflammatory phenotype through IL‐4‐incorporated GelMA hydrogels via integrin α2β1 attachment.^[^ ^94^ ^]^

The use of fibrin‐based hydrogels has emerged as a potential biomaterial for improving dermal wound healing. As key immunomodulatory agents, fibrin and fibrinogen regulate the wound environment. As a provisional matrix, fibrin skillfully manages the cytokine balance and controls the transition from inflammatory to anti‐inflammatory responses.^[^ ^95^ ^]^ This intricate modulation accelerates the trajectory of wound healing, driving it from the initial inflammatory phase through proliferation to the final remodeling stage. In the investigation conducted by Hsieh et al., macrophages cultured on fibrin gels and exposed to soluble fibrinogen demonstrated distinct patterns in cytokine secretion.^[^ ^96^ ^]^ Notably, fibrinogen evoked a dual response, stimulating the production of both TNF‐α and IL‐10. Interestingly, when subjected to additional stimulation with IL‐4/IL‐13 or LPS/IL‐4/IL‐13, the effects on cytokine secretion were minimal. Remarkably, macrophages cultured on fibrin exhibited heightened levels of IL‐10 and diminished levels of TNF‐α, regardless of supplementary stimulation. This finding underscores the unique immunomodulatory properties of fibrin in shaping macrophage responses and highlights its potential to influence the cytokine milieu during wound healing.

**Figure 8:**
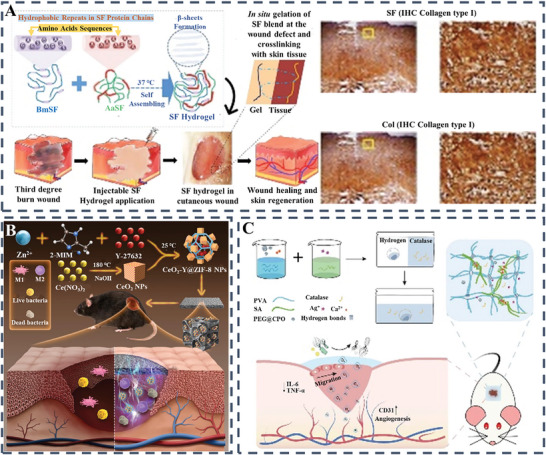
A) A diagram illustrates the process by which a blend hydrogel made of silk fibroin (SF) is formed at body temperature to be utilized in the treatment of wounds. Reproduced with permission.^[^ ^99^ ^]^ Copyright 2018, John Wiley and Sons Ltd; B) Illustrative diagram of CeO_2_‐@ZIF‐8@Gel's compositional elements and assembly procedure, alongside the therapeutic mechanism for healing contaminated DFUs. Reproduced with permission.^[^ ^100^ ^]^ Copyright 2024, KeAi Communications Co; C) PCPS‐gel is engineered to enhance diabetic wound healing by promoting cell migration, and angiogenesis while reducing inflammation and bacterial infection. Reproduced with permission.^[^ ^101^ ^]^ Copyright 2024, Wiley.

Lately, silk fibroin has also been proven to facilitate the emission of IL‐10 from peripheral blood mononuclear cells (PBMCs).^[^ ^97^ ^]^ Fascinatingly, silk fibroin has a built‐in capacity to speed up the injury mending through NF‐κβ signaling pathways. Given this, comprehensive research has been conducted across a range of injuries to oversee the healing of wounds utilizing silk fibroin. This encompasses hypertrophic scars and DFUs where hydrogels derived from silk fibroin initiated specific phases of the wound healing cycle.^[^ ^98^ ^]^ Recently, researchers led by Chouhan have illustrated the application of an injectable hydrogel derived from silk fibroin for the management of severe burns or persistent wounds (**Figure** **8**A).^[^ ^99^ ^]^ The crafted hydrogel was noted to facilitate the therapy for wounds with complete tissue depth. Furthermore, in vivo investigations have validated the potent function of silk fibrin‐based hydrogel in hastening the progression from the inflammatory to the proliferative phase, as indicated by the TNF‐α levels. Significantly, the silk fibroin hydrogel markedly improved the restructuring of the collagen matrix in comparison to the collagen‐based hydrogel. This research indicated the promising healing influence of silk fibrin in the treatment of thermal injuries.

The latest innovative nanomedicine developed by the researchers incorporates the Rho‐associated protein kinase inhibitor Y‐27632 encapsulated in a cerium oxide‐loaded zeolitic imidazolate framework‐8 (CeO_2_‐Y@ZIF‐8) to support immune modulation by mitigating oxidative damage in mitochondria. A novel nanodrug embedded in a photocross‐linkable hydrogel matrix (GelMA) enriched with cationic quaternary ammonium salt groups for antibacterial effects (CeO_2_‐Y@ZIF‐8@Gel) effectively harnesses the superoxide dismutase and catalase‐like functions of CeO_2_ to neutralize excessive ROS and mitigate oxidative stress in mitochondria. Concurrently, Y‐27632 aids in mending damaged mitochondrial DNA, thereby promoting endothelial cell proliferation. Once internalized by endothelial cells, CeO_2_‐Y@ZIF‐8 nanostructures decompose peroxides into water and oxygen both in the cytoplasm and in the mitochondria, resulting in the suppression of the NLRP3 inflammasome pathway and a reduction in the release of oxidatively damaged mitochondrial DNA through the mitochondrial permeability transition pore (mPTP). Moreover, the suppression of the cGAS‐STING pathway activated by impaired mitochondrial DNA encourages M2 phenotype polarization and enhances the production of anti‐inflammatory factors. The CeO_2_‐Y@ZIF‐8@Gel composite effectively orchestrates the intricate relationship between macrophage reprogramming and the promotion of new blood vessel formation, thereby mitigating inflammatory responses in the local environment and promoting a faster healing process (Figure 8B).^[^ ^100^ ^]^ Looking ahead, the development of such nanoparticles holds promise for improving diabetic wound treatment by targeting the intricate interplay between immune responses and mitochondrial repair mechanisms. This innovative approach not only addresses the root causes of delayed wound healing but also offers the potential for enhancing therapeutic outcomes through the modulation of inflammatory pathways and cellular processes. By reshaping the immune microenvironment and promoting tissue regeneration, the resultant represents a progressive strategy with significant implications for advancing wound care and improving patient outcomes in nanomedicine. Interestingly, a cutting‐edge hydrogel patch, PCPS‐gel, designed specifically for diabetic wound treatment, has recently been introduced. This innovative patch combines polyethylene glycol‐modified calcium peroxide (PEG@CPO) microparticles and silver ions (Ag^+^) within a dual‐network hydrogel matrix comprising PVA and sodium alginate (SA). By incorporating catalase to mitigate the harmful effects of ROS, PCPS‐gel is packaged in a convenient kit with catalase‐infused sterile water and hydrogel. The sustained release of oxygen for up to 7d by PCPS‐gel autonomously supports cell migration and angiogenesis in low oxygen conditions, while also promoting ECM synthesis, angiogenesis, and reducing inflammatory cytokine levels. With an impressive 84.2% closure rate for diabetic wounds by the 7th day, PCPS‐gel surpasses existing oxygen‐releasing agents, marking a significant advancement in wound healing technology with promising implications (Figure 8C).^[^ ^101^ ^]^

### 4.2 Hydrogel Surface Characteristics for Immune System Regulation

**Figure 9:**
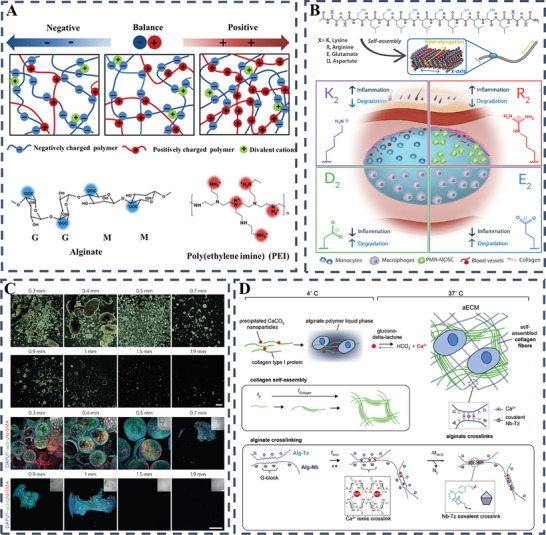
A) Hydrogel formation through the assembly of oppositely charged alginate and branched PEI polyelectrolytes, featuring distinct chemical structures for ionic complexation. Reproduced with permission.^[^ ^103^ ^]^ Copyright 2018, American Chemical Society; B) Multidomain Peptides (MDPs) self‐assemble into biomaterials with tunable immune responses; anionic MDPs induce low inflammation, cationic lysine variants elicit mild reactions, while arginine ones provoke stronger responses with PMN‐MDSC, angiogenesis, and collagen deposition. Reproduced with permission.^[^ ^104^ ^]^ Copyright 2020, Elsevier; C) Alginate spheres of varying sizes were implanted in mice to study fibrosis; larger spheres showed less cellular overgrowth. Bimodal scaffolds with 60‐µm channels and 30‐µm spherical pores were uniformly seeded with cardiomyocytes, as shown by SEM and digital imaging. Reproduced with permission.^[^ ^108^ ^]^ Copyright 2015, Nature Portfolio; D) Implanted alginate spheres in mice showed fibrosis inverse to size, while chick cardiomyocyte‐seeded bimodal scaffolds demonstrated cellular gradient, preferential channel residency, and viability, with hESC‐CM scaffolds exhibiting high β‐myosin density, troponin T expression, and cell distribution via live/dead assay. Reproduced with permission.^[^ ^112^ ^]^ Copyright 2010, National Academy of Sciences.

The intricate modulation of immune responses, encompassing the nuanced polarization of macrophages, is intricately linked to the precise manipulation of hydrogel surface properties. Notably, the surface chemistry of hydrogels emerges as a key determinant in orchestrating immune reactions. While the significance of surface topography in biomaterials is acknowledged as critical in influencing immune responses, particularly macrophage polarization, comprehensive mechanistic investigations into these immunomodulatory effects remain an uncharted territory thorough exploration.^[^ ^102^ ^]^ The intricate interplay of physicochemical attributes in synthetic polymer‐based hydrogels, with a particular emphasis on electrical charge, exerts a critical influence on the spectrum of pro‐inflammatory responses they evoke. Variables such as chirality, size, stiffness, and porosity collectively contribute to the complex landscape of inflammatory reactions incited by these hydrogels. In an investigation conducted by Zhang and collaborators, the strategic manipulation of the ratio between negatively charged alginate and positively charged poly (ethylene imine) within polyelectrolyte hydrogels emerged as a potent strategy in attenuating foreign body reactions and inhibiting the formation of collagen. In a state of electrical neutrality, the hydrogels showcased remarkable antifouling characteristics, effectively warding off proteins, bacteria, and cells, and maintaining capsule‐free integrity even three months post‐subcutaneous implantation. Immunofluorescent staining indicated a notable reduction in macrophage migration toward the interface between the hydrogels and surrounding tissue, underscoring the hydrogels’ ability to modulate immune responses and promote long‐term compatibility (**Figure** **9**A).^[^ ^103^ ^]^ In a fascinating study, Hartgerink et al. investigated the early inflammatory responses induced by multidomain peptide hydrogels incorporating groups such as amines, guanidine's or carboxylic acids. Despite sharing similar self‐assembled nanofibrous structures and rheological characteristics, hydrogels with a negative charge triggered minimal inflammatory reactions. These responses were characterized by reduced infiltration of macrophages, decreased production of inflammatory cytokines, and the notable absence of collagen deposition or blood vessel formation within the hydrogels. In contrast, the arginine‐enriched hydrogel induced a robust inflammatory reaction, as indicated by the sustained presence of polymorphonuclear myeloid‐derived cells even ten days post‐implant (Figure 9B).^[^ ^104^ ^]^ In a recent study, researchers investigated the influence of a GelMA hydrogel with a variety of microscale patterns, such as micropatterns, micropillars and microgrooves – on the behavior of human macrophages. Both conventional markers and gene expression profiles were assessed. The results indicated unique gene expression patterns in macrophages cultured on microgrooves and micropillars, particularly genes associated with initial metabolic activities. Despite these variations in gene expression, the standard phenotyping methods related to surface marker expression remained consistent across different conditions. Recent research highlighted the significance of surface chemistry in immune responses, suggesting that hydrophilic surfaces with a neutral charge led to reduced macrophage adhesion compared to hydrophobic and charged surfaces.^[^ ^105^ ^]^ In a study by Jiang et al., the efficacy of zwitterionic hydrogels in preventing capsule formation and enhancing microvessel growth in the adjacent tissue of mice over 3 months was demonstrated. These hydrogels, composed of poly(carboxy betaine methacrylate) (PCBMA) synthesized from a CBMA and a carboxy betaine cross‐linker (CBMAX), displayed reduced inflammation, increased blood vessel density, and a more evenly distributed collagen structure in the surrounding tissue after being implanted in mice for 3 months. In contrast to PHEMA hydrogels, PCBMA hydrogels showed a greater attraction to macrophages producing anti‐inflammatory markers such as arginase‐1 and IL‐10, and a reduced presence of macrophages expressing pro‐inflammatory markers such as IL‐12, IL‐1R1, iNOS and TNF‐𝛼.^[^ ^106^ ^]^

Scumpia and his research team noted that the D‐peptide crosslinked microporous annealed particle (MAP) hydrogel incited targeted immune responses to specific antigens, leading to the rejuvenation of skin, activation of hair follicles, and enhancement of tissue tensile strength. The hydrogel microbeads, synthesized in L‐ or D‐configuration, were fabricated using four‐arm PEG‐vinyl sulfone and L‐ or D‐MMP‐sensitive peptides. When administered subcutaneously in mice, the d‐MAP formulation increased the number of IL‐33^+^F4/80^+^ macrophages around the implants and induced a T‐cell driven immune response that produced IgG1 and IgG2a antibodies. In a murine model with splinted excisional wounds, treatment with D‐MAP or an equivalent L/D‐MAP ratio resulted in minimal or absent residual hydrogel, leading to increased dermal thickness, improved hair follicle regeneration, and enhanced skin tensile strength within a timeframe.^[^ ^22^ ^]^ Moreover, different physical properties of hydrogels, such as size, stiffness, and porosity, can also impact the host immune responses.^[^ ^107^ ^]^ In an intriguing study, Anderson and colleagues examined the influence of alginate hydrogel size and morphology on the immune response following implantation in rodents and non‐human primates (Figure 9C).^[^ ^108^ ^]^ They introduced Ba^2+^‐crosslinked alginate hydrogel spheres of various sizes (ranging from 0.3 to 1.9 mm) into the intraperitoneal cavities of mice for 14d. As sphere size increased, there was a marked reduction in the accumulation of proteins such as F‐actin, α‐smooth muscle actin, CD68, TGF‐*β*, collagen 1a1 and collagen 1a2. In primate models, the 1.5 mm beads were still translucent at 14 and 28d after implantation, demonstrating reduced cellular accumulation and myofibroblast response associated with fibrosis, in contrast to the smaller 0.5 mm beads. Rat pancreatic islet cells enclosed in 1.5 mm capsules‐maintained blood glucose levels for a remarkable 175d in streptozotocin‐induced diabetic mice, a duration five times longer than that observed with islets in 0.5 mm capsules.

The stiffness of hydrogels is a key factor in controlling a variety of cellular processes, including those related to the immune.^[^ ^108^ ^]^ O'Brien and his colleagues illustrated how the stiffness of polyacrylamide gels at varying levels (11, 88, and 323 kPa) influenced the polarization, functionality, and migratory behavior of macrophages. Particularly on the rigid (323 kPa) collagen‐coated polyacrylamide gels, macrophages from THP‐1 cells displayed a transition towards a pro‐inflammatory phenotype, a decrease in their phagocytic capacity, and exhibited a slow mesenchymal migration that relied on podosomes and was not influenced by RhoA.^[^ ^109^ ^]^ The study conducted by Wang and his team highlighted the significant impact of methacrylate‐gelatin hydrogel stiffness (ranging from 2 to 29 kPa) on macrophage phenotype in vitro and the inflammatory response in vivo. Notably, when macrophages were cultured on firmer hydrogels, they displayed a tendency toward a pro‐inflammatory phenotype, characterized by increased cell spreading, elevated levels of F‐actin and focal adhesion staining, and enhanced secretion of pro‐inflammatory cytokines. Following implantation in mice, the stiffer hydrogels exhibited a notable decrease in macrophage infiltration and the formation of a thicker fibrous capsule. This emphasizes the crucial role of hydrogel stiffness in influencing macrophage behavior and tissue responses.^[^ ^110^ ^]^

The dimensions of a scaffold's pores are known to be important determinants of macrophage and host immune responses.^[^ ^111^ ^]^ Ratner et al. have developed a specialized hydrogel for cardiac tissue regeneration with a network of spherical pores and elongated channels. This novel hydrogel, composed of poly(2‐hydroxyethyl methacrylate‐co‐methacrylic acid), was attentively created by targeted photocross‐linking of 2‐hydroxyethyl methacrylate and methacrylic acid. The creation of these hydrogels involved the deliberate incorporation of sacrificial constructs, including PMMA particles and a PC center surrounded by a PMMA optical fiber coating. After a thorough 5d dissolution period in dichloromethane to remove the PC/PMMA templates, the hydrogel was rehydrated and then subjected to a refined surface modification using collagen I. When placed in the myocardium of athymic nude and immunocompetent rats, these innovative hydrogels showed a marked ability to induce macrophage polarization toward the M2 phenotype, leading to significant neovascularization (Figure 9D).^[^ ^112^ ^]^

### 4.3 Stem Cells‐Mediated Immune Modulation

Biomaterials that modulate immunity have shown promise in tissue repair, particularly in minimizing scarring. This is achieved by targeting MSCs or ASCs to the site of injury. Endowed with a broad spectrum of immunomodulatory functions, these cells are adept at orchestrating different immune responses, both adaptive and innate. The therapeutic efficacy of MSCs and ASCs is largely mediated through the activation of paracrine signaling pathways, providing a regenerative approach to healing.^[^ ^113^ ^]^ MSCs secrete a plethora of bioactive molecules that are critical for tissue repair, including anti‐inflammatory cytokines, chemotactic chemokines that direct cell migration, proliferative growth factors, and regulatory hormones that control physiological functions. Consistent with the hydrogel‐based strategies discussed previously, these molecular interventions are often aimed at counteracting the pro‐inflammatory effects of TNF‐α, thereby supporting the overall healing mechanism.^[^ ^114^ ^]^ In this analysis, we've delved into an array of regulated delivery methods that impact the various stages of the wound healing continuum, spanning from the inflammatory phase to complete restoration. This encompasses the strategic deployment of a diverse spectrum of bioactive elements, including but not limited to cytokines, growth factors, genetic material, immunoglobulins, and additional therapeutic agents. A summary of these strategies and their respective outcomes can be found in **Table** **1**. Additionally, MSCs emit extracellular vesicles‐nanoscale particles rich in nucleic acids, lipids, and proteins‐that actively combat cell death, tweak immune reactions, and encourage neoangiogenesis, the creation of new blood vessels.^[^ ^115^ ^]^ Consider the MSC‐conditioned medium, teeming with bioactive soluble factors, notably PGE2. PGE2 actively suppresses the secretion of pro‐inflammatory cytokines and concurrently stimulates an upsurge in the levels of anti‐inflammatory cytokines and TGF‐β1. This prostaglandin also plays a pivotal role in curtailing T cell proliferation rates within the injured area. Moreover, PGE2 aids in modulating the immune response by nudging the balance from Th1 to Th2 cells, which in turn, bolsters the body's efforts to quell inflammation and enhances the trajectory of tissue repair and restoration.^[^ ^116^ ^]^ The immunoregulatory capabilities of MSCs underscore their potential application as therapeutic agents for managing autoimmune disorders and healing chronic wounds.^[^ ^117^ ^]^ Recently, Blázquez and colleagues leveraged the immunomodulatory attributes of MSCs to fabricate MSC‐impregnated meshes, akin to readily available commercial products. These engineered meshes exhibited a modest capacity to steer macrophages toward an anti‐inflammatory phenotype.^[^ ^118^ ^]^ Furthermore, the co‐culture of MSCs with macrophages led to the differentiation of macrophages into a regulatory phenotype. This was evidenced by a reduction in the levels of TNF‐α and IL‐12, accompanied by an upregulation of IL‐10 expression.^[^ ^116^ ^]^

**Table 1:** Comparative analysis of hydrogel‐based immunomodulation strategies for chronic wound healing: merits and limitations.

Biological molecules	Category	Composition	Merits	Demerits	Ref.
Cytokines	IL‐10	Dextrin, Vinyl Methacrylate, and SC16	Enhanced Stability, Controlled Release, Biocompatibility; Simple Preparation	Initial Burst Release; Release Profile Dependency; Potential for Incomplete Release; Long‐Term Stability	[130c]
	IL‐10	Collagen, polyethylene‐eimine ‐DNA complexes (SiNP), and 3T3 fibroblasts	Sustained release; Local delivery; Cell integration; Reduced cytotoxicity; Spatio‐temporal control;	Complex fabrication; Limited by macrophage phenotype; Need for further research;	[130d]
	MCP‐1	Multidomain peptides and β‐sheets	Biphasic release pattern; Biomimetic properties; Shear stress recovery; Promotion of M2 Macrophages	Potential cytokine loss; Complex interactions; Species‐specific responses; Inflammatory environment	[132]
Antibody	Anti‐TNF‐αantibody	Chitosan	Sustained release; Versatility; Local drug delivery; Thermosensitive gelation	Complex release kinetics; Potential Interactions with Therapeutics; Clinical feasibility	[133]
	Anti‐TNF‐α antibody	Low molecular weight glycosyl‐nucleoside	Shear‐mediated release; Reversible sol‐gel transitions; Stability and biocompatibility; Ease of Administration	Weak hydrogel structure; Temperature sensitivity; Diffusion coefficient variability	[134]
	Anti‐TNF‐α antibody	HA conjugation on Anti‐TNF‐α	Sustained local delivery; Reduced inflammation; Improved healing outcomes	Complex synthesis; Potential for immune response; Limited duration of action	[135]
Anti‐inflammatory factor	Resolvin D1	Chitosan Scaffolds	Decreased inflammatory response; Polarization to M2 macrophages; Reduced fibrous capsule formation	Complex delivery system; Potential variability in responses; Lack of long‐term studies	[136]
	Catechol	Chitosan, Iron and Oxidized HA	High tissue adhesion Strength; In situ controlled and sustained release	Complex synthesis; Potential cytotoxicity concerns	[137]
	Peptide and GM‐CSF	Alginate, GM‐CSF; PLG microparticles and Au NPs	Localized delivery; Modulation of immune cells; Induction of antigen‐specific Tregs	Inflammatory response to PLG microparticles; Potential for T Cell activation	[138]

**Figure 10:**
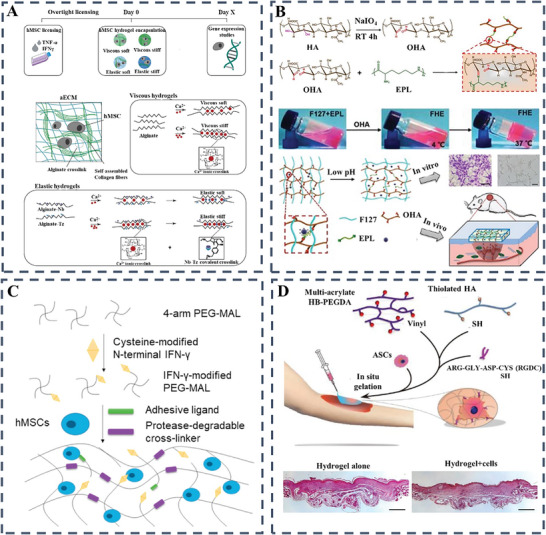
A) The aECM hydrogel system, with its multi‐tiered and modifiable covalent and ionic crosslinking, integrates Type I collagen proteins (green) with calcium carbonate nanoparticles (red) to form a consistent suspension at 4 °C. Reproduced with permission.^[^ ^119^ ^]^ Copyright 2019, Elsevier BV; B) Injectable FHE hydrogel features multifunctional synthesis utilizing oxidized HA and ε‐poly‐L‐lysine, with a thermally induced sol‐gel transition evident in its double‐network structure. Reproduced with permission.^[^ ^121^ ^]^ Copyright 2019, Ivyspring International Publisher; C) Attaching cys‐IFN‐γ to PEG‐4MAL hydrogels and the degradation‐triggered release. Reproduced with permission.^[^ ^123^ ^]^ Copyright 2019, Elsevier BV; D) Diagram of ASC encapsulation in a hydrogel cross‐linked by hyperbranched polyPEGDA, HA‐SH, and RGDC peptides via Michael addition. Reproduced with permission.^[^ ^124^ ^]^ Copyright 2020, Elsevier BV.

Pioneering research by Mooney and colleagues has shed light on the intriguing interplay between the mechanical properties of hydrogels and the immunomodulatory functions of MSCs. Their findings have elucidated that the viscoelastic nature and rigidity of the hydrogel scaffold can significantly influence the expression of key immunomodulatory markers within MSCs (**Figure** **10**A).^[^ ^119^ ^]^ Recent research has uncovered that the regenerative potential of transplanted MSCs may be accompanied by considerable concerns regarding their susceptibility to microbial adhesion, particularly from bacteria like Staphylococcus aureus, as well as the bacteria's ability to form colonies or biofilms.^[^ ^120^ ^]^ Encapsulating MSCs within an appropriate hydrogel can significantly inhibit the colony‐forming capabilities of free‐floating Staphylococcus aureus (S. aureus), while still maintaining the multipotency of the MSCs. Recently, an advanced self‐healing and shear‐thinning hydrogel, fortified with exosomes from adipose‐derived mesenchymal stem cells (AMSCs‐exo), has been engineered for skin repair. This hydrogel was synthesized via a Schiff base reaction between oxidized hyaluronic acid (OHA) and poly‐ε‐L‐lysine (EPL), enhanced by the incorporation of Pluronic F127 (yielding F127/OHA‐EPL). The injectable hybrid hydrogel, enriched with AMSCs‐exo, demonstrated enhanced ease of injection, which significantly advanced the healing process of full‐thickness diabetic skin wounds. (Figure 10B).^[^ ^121^ ^]^ Another injectable hydrogel formulation was crafted by leveraging the interaction between highly branched, multi‐acrylated polyethylene glycol (PEG) macromers and thiol‐modified HA (HA‐SH), tailored for the purpose of adipose‐derived stem cell (ADSC) therapy. This hydrogel demonstrated remarkable mechanical properties and resistance to biofouling. Furthermore, the hydrogel enriched with adipose‐derived stem cells (ADSCs) exhibited promising regenerative potential, particularly notable for its targeted applications.^[^ ^122^ ^]^

The hydrogel‐mediated concurrent delivery of cells and signaling molecules like growth factors or cytokines is emerging as a potent method for addressing a spectrum of chronic ailments and wounds. For instance, to tackle the obstacles in stem cell administration for managing inflammatory bowel diseases, Garcia and colleagues engineered an injectable hydrogel intended to foster immunomodulatory activities. This gel formulation was crafted using a 4‐armed PEG macromer (PEG‐4MAL) featuring maleimide functionality, coupled with recombinant IFN‐γ and human mesenchymal stem cells (hMSCs). Notably, this composite hydrogel displayed augmented cytokine production and expedited the healing process of colonic mucosal wounds in both immunosuppressed and immunologically capable mice (Figure 10C).^[^ ^123^ ^]^ Such a designed platform has significant potential for the clinical translation and efficacy of hMSC‐based therapies.

In a recent study, a hydrogel was developed to encapsulate ADSCs using HA‐bis(PEG)diacrylate (HB‐PEGDA), HA‐SH and RGD peptides. The inclusion of RGD peptides effectively altered cell behavior and increased the release of pro‐angiogenic signals. When applied to wounds, these hydrogels not only promoted the growth of new blood vessels, but also accelerated the healing process and reduced scarring (Figure 10D).^[^ ^124^ ^]^

**Figure 11:**
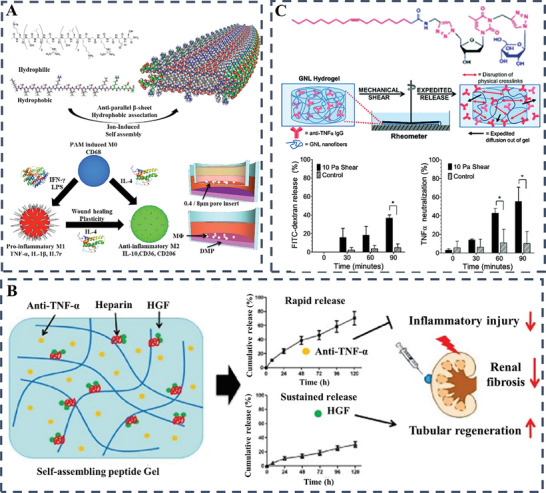
A) Ion‐induced self‐assembly of “SLac” peptides, culminating in the formation of extensive nanofiber networks. Reproduced with permission.^[^ ^132^ ^]^ Copyright 2015, Wiley‐VCH; B) Illustration of SAP/Hep hydrogel for dual delivery of anti‐TNF‐α and HGF. Reproduced with permission.^[^ ^144^ ^]^ Copyright 2019, Elsevier BV; C) Schematic of rheometer‐induced release where “Y” denotes entrapped macromolecules, slow to disperse into the depicted light blue water bath, constrained by blue GNL nanofiber networks. Reproduced with permission.^[^ ^134^ ^]^ Copyright 2016, Université Laval.

Among the many bioactive components, the genetic codes for growth factors and cytokines stand out as highly effective in promoting wound closure. Consequently, the inclusion of cytokines in treatment plans has become a novel approach due to their vital role at every stage of the wound healing process.^[^ ^125^ ^]^ The most recent high‐priority and powerful clinical strategy to reduce scarring is based on the pioneering use of specific bioactive agents: Transforming Growth Factor‐beta 3 (TGF‐β3), Mannose‐6‐phosphate (M6P), Interleukin‐10, known as Prevascar, combined with the anti‐inflammatory drug Nefopam, sold as ScarX, this strategy represents a significant advance in the field of scar reduction therapies.^[^ ^126^ ^]^ Among the many cytokine‐targeted strategies, targeting local IL‐10 levels has emerged as a common method of treating chronic wounds. This approach is favored because IL‐10 has significant effects on a wide range of immune cells. In addition, IL‐10 promotes the growth of Tregs, acts as a potent anti‐inflammatory cytokine, and aids in the reprogramming of macrophage activity, which in turn accelerates wound healing.^[^ ^127^ ^]^ IL‐10 is known to stimulate a wide range of immune cells, including T and B lymphocytes and mast cells, while also exerting an immunosuppressive effect on monocytes and macrophages, thus playing a role in the regulation of immune responses.^[^ ^128^ ^]^ In addition, IL‐10 inhibits the generation of a diverse panel of cytokines (including IL‐1, IL‐2, IL‐3, IL‐6, IL‐8, IL‐12, TNF and IFN) and modulates the expression of CD86 downregulation, culminating in the suppression of antigen presenting capabilities.^[^ ^129^ ^]^ Using the sustained immunomodulatory properties of IL‐10, recent studies have highlighted its ability to suppress excessive immune responses. As a result, there is growing support for the clinical use of IL‐10, either alone or in combination with other biological agents, to finely regulate the activity of the immune system.^[^ ^130^ ^]^ Beyond IL‐10, the cytokine landscape for immunomodulatory therapies is full of key players. IL‐4, for example, has emerged as a key orchestrator in the immune symphony, demonstrating a remarkable ability to catalyze the transition from pro‐inflammatory to pro‐reparative responses. This cytokine not only acts as a conductor, orchestrating the rhythm of the immune system, but also as a potent stimulator of the proliferation of cells with anti‐inflammatory properties, thereby creating an environment conducive to tissue healing and immune balance.^[^ ^131^ ^]^ Kumar and colleagues have masterminded the development of a frontier self‐assembling peptide‐heparin composite hydrogel that precisely choreographs a two‐step cytokine release strategy for IL‐4 and MCP‐1, elegantly illustrated in **Figure** **11**A.^[^ ^132^ ^]^ MCP‐1, a potent chemokine released from the depths of activated monocytes, plays a key role in amplifying inflammatory triggers through the synthesis of ROS and nitrogen species (ROS/RNS). This innovative study demonstrates that the effective interaction between the fibrous structure of the hydrogel and macrophages, coupled with a two‐phase cytokine release strategy, results in robust activation of THP‐1 monocytes and macrophages, promoting a marked shift toward an anti‐inflammatory phenotype.

Besides, the in situ gelation of the hydrogel offers the ability to retain cytokines for potential release in response to various cellular immune triggers. In addition to the cytokines already considered clinically viable, others with significant potential, such as interleukin‐1 beta (IL‐1β) and stromal cell‐derived factor‐1 alpha (SDF‐1α), are being actively investigated for their role in the treatment of chronic wounds. For example, IL‐1β is part of an inflammatory feedback loop that maintains a macrophage phenotype conducive to persistent inflammation, which aids the healing process of wounds associated with diabetes. Recent research has shown that the use of an antibody targeting IL‐1β significantly improved wound healing rates in a diabetic mouse model.^[^ ^139^ ^]^

The key target in the strategic regulation of antibody delivery is the neutralization of TNF‐α’s influence. Extensive evidence indicates that the hyperglycemic state in diabetes promotes a macrophage phenotype that is highly inflammatory. This pro‐inflammatory condition is linked to the excessive release of TNF‐α, which is harmful to the progression of keratinocyte movement.^[^ ^140^ ^]^ As a result, the specific inactivation of TNF‐α where the injury occurs can markedly promote the recovery of persistent wounds. This improvement is realized by curtailing the influx of cells that cause inflammation and by restraining the proliferation of macrophages exhibiting a pro‐inflammatory response.^[^ ^141^ ^]^ Research conducted in a rodent model revealed that the administration of an anti‐TNF‐α antibody caused heightened inflammation. This intervention disrupted the equilibrium between pro‐inflammatory and anti‐inflammatory traits, transitioning the macrophage's phenotype toward a more anti‐inflammatory stance.^[^ ^142^ ^]^ Considering this, diverse medicinal compositions have been created and made accessible for purchase to curb the emission of TNF‐α. This includes treatments like infliximab and golimumab, which have been examined in additional literature.^[^ ^143^ ^]^ Injecting antibodies designed to block TNF‐α could lead to harmful side effects. As far as we know, only a few studies have focused specifically on the controlled release of such anti‐TNF‐α antibodies.^[^ ^133^, ^134^, ^135^ ^]^ In addition, controlled release mechanisms for anti‐TNF‐α have largely been studied in inflammatory settings, with little attention paid to their impact on the balance of macrophage polarization toward pro‐inflammatory or reparative states. In the latest research, a self‐assembling KLD2R peptide/heparin hydrogel has been formulated to co‐deliver anti‐TNF‐α and hepatocyte growth factor (HGF), with a controlled release profile aimed at mitigating injury resulting from ischemia‐reperfusion (I/R) events (Figure 11B).^[^ ^144^ ^]^ In a separate investigation, Kaplan and colleagues created nanofibers composed of low molecular weight glycosylnucleoside‐lipid amphiphiles that exhibit remarkable shear‐thinning properties (Figure 11C).^[^ ^134^ ^]^ These engineered hydrogels, when subjected to mechanical shear, modulate the release of anti‐TNFα for the treatment of autoimmune diseases. However, the precise impact of anti‐TNF‐α on immune cell dynamics remains largely unexplored. Despite significant recent advances in antibody delivery systems for immune modulation, continued refinement and research are essential to facilitate their transition into clinical practice.

In addition to a variety of biological elements, a number of small molecule‐based immunomodulators – such as resolvin D1 and lipoxin A4 – have demonstrated efficacy in wound healing. These substances act at multiple stages of the wound healing cascade, particularly modulating the inflammatory phase. They do this by promoting polarization toward an anti‐inflammatory phenotype, enhancing antigen‐specific CD4^+^ T cell responses and reducing the expression of IL‐1β.^[^ ^136^, ^145^ ^]^ Regulatory T cells (Tregs) in mice skin suppress autoimmunity and reduce inflammation post‐wounding, with increased presence at wound sites aiding healing.^[^ ^146^ ^]^ Their absence slows down healing and increases inflammation.^[^ ^147^ ^]^ Tregs may also play a role in scar formation, as seen with GATA3 removal leading to more fibrosis.^[^ ^148^ ^]^ For example, in one research project, catechol, which is essential for the adhesive properties of mussels, was used to produce IPN hydrogels. In this study, self‐covalent bonding between chitosan and deoxidized HA was incorporated and the hydrogel structure was further strengthened with trivalent iron Fe^3+^ ions.^[^ ^137^ ^]^ The resulting hybrid hydrogel demonstrated robust attachment to moist biological tissues and effectively reduced the release of pro‐inflammatory cytokines such as IL‐1β. In addition, the in vivo studies indicated rapid vascularization following application of these IPN hydrogels. The integration of IPN hydrogels with growth factors has been explored to modulate immune responses. For example, Verbeke and colleagues investigated the simultaneous delivery of granulocyte‐macrophage colony‐stimulating factor (GM‐CSF) with the BDC peptide. This was achieved by using an injectable gel that creates pores to facilitate the release of these bioactive agents.^[^ ^138^ ^]^ In a mouse model of type 1 diabetes, the BDC peptide, a peptide antigen mimic, is identifiable by T cells.^[^ ^149^ ^]^ For this research, an alginate‐derived hydrogel served as a protective shell for GM‐CSF‐bound gold nanoparticles (Au NPs) and for the encapsulation of peptides within PLGA microspheres. The subcutaneous delivery of this composite hydrogel facilitated a focused expression of antigen‐specific T cells within the lymphatic nodes. As a result, T cells that are specific to the antigen, particularly the CD4^+^ variety, have increased the concentration of the corresponding antigen. These cells also exhibit markers characteristic of regulatory Tregs, which contribute to a reduction in the rate of disease progression in non‐obese diabetic mice.^[^ ^138^ ^]^ Nonetheless, the simultaneous delivery of multiple substances with varying degrees of water and oil solubility was not achievable using a single hydrogel matrix.

To address the challenge of delivering both water‐soluble and fat‐soluble substances simultaneously, a novel dual‐affinity hydrogel was crafted from PEGDA. This innovative material incorporated the growth factor‐binding capacity of a heparin‐related compound (Hep‐N) and the lipid‐escorting function of albumin.^[^ ^150^ ^]^ The hydrogel efficiently encapsulated and later released two key bioactive agents, SDF‐1α and the S1PR3 receptor, without compromising their bioactivity. When applied to skin injuries, the hydrogel facilitated a swift shift in macrophage behavior and the formation of new blood vessels, thereby hastening the tissue healing process.

## 5 Insights and Outlook: Conclusions and Future Perspective

Hydrogels are transforming immunomodulation and skin regeneration in combination with advanced manufacturing methods, including 3D printing. Smart, flexible wound dressings and bioelectronic interfaces with biological tissues are emerging as dominant innovations in chronic wound care. These hydrogel‐based bioelectronics offer dual functionality: real‐time wound monitoring and controlled bioactive molecule release, heralding a new era in smart drug delivery systems. A recent innovative includes a conductive hydrogel composite of polydopamine‐functionalized silver nanoparticles, polyaniline, and PVA, designed for epidermal sensors and diabetic wound dressings. Yet, there remains a knowledge gap in the development of hydrogel‐based bioelectronics capable of precisely controlling the release of immunomodulatory agents, a challenge that is central to advancing chronic skin wound therapies. Innovative biomedical research is illuminating the unparalleled potential of targeted immunological intervention to expedite chronic wound recovery. Novel methodologies are being well‐crafted charted for the precise temporal and spatial orchestration of immune responses. The ascendance of immunomodulatory scaffolds heralds the genesis of revolutionary approaches to achieving scarless tissue regeneration. This exhaustive review delves into the sophisticated engineering of hydrogels, ingeniously endowed with bespoke characteristics to regulate immune responses and expedite the closure of persistent wounds. Emerging data suggests that these hydrogels’ intrinsic attributes can proficiently direct both the innate and adaptive immune cell responses across the wound healing continuum. The current academic vogue is centered on the innovation of hydrogel synthesis protocols to adeptly navigate adaptive immunity. Hence, a dissection of the interplay between immune cells and the spectrum of natural to synthetic hydrogels is essential for unveiling profound biological mechanisms. Nevertheless, the clinical integration of these innovative biomaterials is contingent upon a stringent evaluation of their in vivo performance.

Hydrogels, crafted for chronic wound immunomodulation, are a cornucopia of bioactive, ranging from antimicrobials to growth factors and cells. They serve as an impenetrable bulwark against proteolysis, with the intricate challenge of controlled bioactive release ingeniously tackled by stimuli‐responsive designs and cutting‐edge micro/nanoscale engineering. A philosophy of innovation, the GelMA‐based microneedle patch, not only facilitates pDNA delivery but also modulates immune responses with precision‐tunable γ‐PGA. The synergistic co‐delivery of a plethora of immunomodulators through these patches heralds an unexplored yet auspicious frontier for skin regeneration.

Hydrogels are at the vanguard of skin regeneration and immunomodulation, synergized by state‐of‐the‐art manufacturing techniques like 3D printing. Pioneering smart wound dressings and bioelectronic interfaces with biological tissues are defining the future of chronic wound care. These hydrogel‐based bioelectronics are a dual‐edged sword: offering real‐time wound surveillance and precise bioactive molecule dispensation, thus inaugurating an epoch of intelligent drug delivery systems. A recent innovation is the conductive hydrogel composite, integrating polydopamine‐functionalized silver nanoparticles, polyaniline, and PVA, tailored for epidermal sensors and diabetic wound management. However, a chasm in knowledge persists regarding the development of hydrogel‐based bioelectronics with the finesse to regulate the release of immunomodulatory agents, a conundrum principal to the evolution of chronic skin wound therapies.

The process of wound healing is complex and dynamic, transcending the scope of conventional clinical care and offering a spectrum of opportunities and challenges. In the initial stages of concept and feasibility, preclinical research is crucial for grasping the fundamental biological aspects linked to the device's intended purpose, guiding its design and development. Researchers are obligated to undergo stringent preclinical assessments to meet regulatory standards and present robust evidence to the FDA, often via an Investigational Device Exemption, before clinical trials can commence. This evidence is vital for a thorough evaluation of wound care interventions, emphasizing safety, efficacy, and practicality. Understanding the FDA's prerequisites for preclinical evidence is key to ensuring regulatory compliance and streamlining the approval process. For medical devices, this involves comprehensive testing, including biocompatibility assessments, biomechanical evaluations, and in vivo studies using animal models that mimic human wound healing dynamics. When it comes to pharmaceuticals, it's imperative to delineate the depth and precision of the preclinical data demanded by the FDA, encompassing aspects like toxicity profiles and pharmacokinetics. With the FDA's shift away from mandatory animal testing for new drug approvals, innovative methodologies such as organ‐on‐chip technology offer promising avenues for assessing the efficacy of prospective wound care solutions. Modern preclinical research strategies, including organ‐on‐a‐chip models, sophisticated imaging technologies, and multi‐omics analyses, have the potential to augment the prognostic accuracy of preclinical studies, hastening the progression of wound care innovations that hold clinical significance.

Looking ahead, a deeper grasp of the molecular underpinnings of immunotherapy facilitated by biomaterials, coupled with advancements in bioengineering, is poised to inspire researchers to craft more potent immunological engineering methods. This progression aims to bridge the divide between foundational research and clinical applications, thereby enhancing the curative capabilities of hydrogels. Nonetheless, a plethora of unresolved challenges and contemplations must be navigated to ensure the seamless clinical integration of hydrogels for the treatment of wound healing:

1. Concerns may be raised regarding the security of immunomodulatory hydrogels, as their potential to excessively dampen the immune system could inadvertently trigger certain immune‐related disorders. Hence, there is a necessity for the development of hydrogels that are designed with a high degree of specificity and precision to proactively circumvent unforeseen health complications.
2. Commonly employed murine and rodent‐based models for diabetic wound healing often fall short in accurately reflecting the human condition, primarily due to their tendency to contract rather than undergo re‐epithelialization. As such, there is a significant need for alternative animal models that can more effectively replicate the intricate pathological milieu of chronic wound in humans, as well as their reactions to various therapeutic interventions. In parallel, the convergence of innovative experimental methodologies, such as the use of organs‐on‐chips technology, may pave the way for a more promising approach to in vivo assay validation.
3. Recent findings suggest that the mobilization of the superficial fascia post‐skin injury significantly influences tissue repair and the development of scars. Upcoming research aims to explore the impact of immunomodulatory hydrogels when used as an ECM scaffold to substitute for the ECM in the wound area derived from the superficial fascia. This approach could potentially offer novel avenues for enhancing the healing process of chronic wound while minimizing scar formation.
4. Due to persistent bacterial infections and irregular immune responses, chronic wounds often show reduced efficacy to treatment. There's an urgent need to develop hydrogels that can regulate immunity, fight infection, promote angiogenesis, aid tissue repair and neutralize ROS. Making these hydrogels cost effective could increase their clinical use in the treatment of chronic wounds.

## Conflict of Interest

The authors declare no conflict of interest.

## References

[1] M. Kharaziha , A. Baidya , N. Annabi , Adv. Mater. 2021, 33, 2100176. 10.1002/adma.202100176 PMC8489436 34251690

[2] Y. Wang , Y. Jiang , G. Ni , S. Li , B. Balderson , Q. Zou , H. Liu , Y. Jiang , J. Sun , X. Ding , Adv. Sci. 2024, 11, e2306703. 10.1002/advs.202306703 PMC11132071 38561967

[3] H. Amani , M. A. Shahbazi , C. D'Amico , F. Fontana , S. Abbaszadeh , H. A. Santos , J. Cont. Release 2021, 330, 185. 10.1016/j.jconrel.2020.12.019 33340568

[4] C. Huang , P. Y. Sun , Y. Jiang , Y. Liu , Z. Liu , S. L. Han , B. S. Wang , Y. X. Huang , A. R. Ren , J. F. Lu , Q. Jiang , Y. Li , M. X. Zhu , Z. Yao , Y. Tian , X. Qi , W. G. Li , T. L. Xu , Nat. Commun. 2024, 15, 5288. 38902277 10.1038/s41467-024-49577-3 PMC11190258

[5] S. Liu , Y. H. Hur , X. Cai , Q. Cong , Y. Yang , C. Xu , A. M. Bilate , K. A. U. Gonzales , S. M. Parigi , C. J. Cowley , B. Hurwitz , J.‐D. Luo , T. Tseng , S. Gur‐Cohen , M. Sribour , T. Omelchenko , J. Levorse , H. A. Pasolli , C. B. Thompson , D. Mucida , E. Fuchs , Cell 2023, 186, 2127. 37098344 10.1016/j.cell.2023.03.031 PMC10321318

[6] O. A. Peña , P. Martin , Nat. Rev. Mol. Cell Biol. 2024, 25, 599. 38528155 10.1038/s41580-024-00715-1

[7] R. Luo , Y. Liang , J. Yang , H. Feng , Y. Chen , X. Jiang , Z. Zhang , J. Liu , Y. Bai , J. Xue , S. Chao , Y. Xi , X. Liu , E. Wang , D. Luo , Z. Li , J. Zhang , Adv. Mater. 2023, 35, 2208395. 10.1002/adma.202208395 36681867

[8] A. Rasmussen , T. Almdal , A. Anker Nielsen , K. E. Nielsen , M. E. Jørgensen , S. Hangaard , V. Siersma , P. E. Holstein , Diabetes Res. Clin. Pract. 2017, 130, 221. 28648855 10.1016/j.diabres.2017.05.025

[9] S. Hangaard , A. Rasmussen , T. Almdal , A. A. Nielsen , K. E. Nielsen , V. Siersma , P. Holstein , Diabetes Res. Clin. Pract. 2019, 151, 177. 31004675 10.1016/j.diabres.2019.04.021

[10] W. Li , Z. Liu , X. Tan , N. Yang , Y. Liang , D. Feng , H. Li , R. Yuan , Q. Zhang , L. Liu , L. Ge , Adv. Healthcare Mater. 2024, 13, e2304365. 10.1002/adhm.202304365 38316147

[11] D. Chouhan , N. Dey , N. Bhardwaj , B. B. Mandal , Biomaterials 2019, 216, 119267. 31247480 10.1016/j.biomaterials.2019.119267

[12] Z. Xie , N. V. Aphale , T. D. Kadapure , A. S. Wadajkar , S. Orr , D. Gyawali , G. Qian , K. T. Nguyen , J. Yang , J Biomed Mater Res A 2015, 103, 3907. 26014899 10.1002/jbm.a.35512 PMC4626281

[13] A. Singh , N. A. Peppas , Adv. Mater. 2014, 26, 6530. 25155610 10.1002/adma.201402105 PMC4269549

[14] S. A. Eming , P. Martin , M. Tomic‐Canic , Sci. Transl. Med. 2014, 6, 265sr6. 25473038 10.1126/scitranslmed.3009337 PMC4973620

[15] Y. Liang , J. He , B. Guo , ACS Nano 2021, 15, 12687. 34374515 10.1021/acsnano.1c04206

[16] Z. Julier , A. J. Park , P. S. Briquez , M. M. Martino , Acta Biomater. 2017, 53, 13. 28119112 10.1016/j.actbio.2017.01.056

[17] J. Larouche , S. Sheoran , K. Maruyama , M. M. Martino , Adv. Wound Care 2018, 7, 209. 10.1089/wound.2017.0761 PMC6032665 29984112

[18] J. Wang , W. Liu , G. Luo , Z. Li , C. Zhao , H. Zhang , M. Zhu , Q. Xu , X. Wang , C. Zhao , Y. Qu , Z. Yang , T. Yao , Y. Li , Y. Lin , Y. Wu , Y. Li , Energy Environ. Sci. 2018, 11, 3375.

[19] W. Zheng , W. Yang , W. Wei , Z. Liu , P. L. Tremblay , T. Zhang , Adv. Healthcare Mater. 2024, 13, e2303138. 10.1002/adhm.202303138 37903562

[20] Z. Zhang , C. He , X. Chen , Adv. Mater. 2024, 36, 2308894. 10.1002/adma.202308894 37909463

[21] X. Zhao , D. Pei , Y. Yang , K. Xu , J. Yu , Y. Zhang , Q. Zhang , G. He , Y. Zhang , A. Li , Y. Cheng , X. Chen , Adv. Funct. Mater. 2021, 31, 2009442.

[22] D. R. Griffin , M. M. Archang , C. H. Kuan , W. M. Weaver , J. S. Weinstein , A. C. Feng , A. Ruccia , E. Sideris , V. Ragkousis , J. Koh , M. V. Plikus , D. Di Carlo , T. Segura , P. O. Scumpia , Nat. Mater. 2021, 20, 560. 33168979 10.1038/s41563-020-00844-w PMC8005402

[23] L. Chávez‐Galán , M. L. Olleros , D. Vesin , I. Garcia , Front. Immunol. 2015, 6, 263. 26074923 10.3389/fimmu.2015.00263 PMC4443739

[24] Y. Xue , X. Yan , D. Li , S. Dong , Y. Ping , Nat. Commun. 2024, 15, 2270. 38491004 10.1038/s41467-024-46210-1 PMC10943244

[25] a) Y. Jiang , S. Sun , Y. Quan , X. Wang , Y. You , X. Zhang , Y. Zhang , Y. Liu , B. Wang , H. Xu , X. Cao , Nat. Commun. 2023, 14, 8455; 38114488 10.1038/s41467-023-43784-0 PMC10730619

[26] a) A. Christofides , L. Strauss , A. Yeo , C. Cao , A. Charest , V. A. Boussiotis , Nat. Immunol. 2022, 23, 1148; 35879449 10.1038/s41590-022-01267-2 PMC10754321

[27] Y. Li , X. Chen , R. Jin , L. Chen , M. Dang , H. Cao , Y. Dong , B. Cai , G. Bai , J. J. Gooding , S. Liu , D. Zou , Z. Zhang , C. Yang , Sci. Adv. 2021, 7, abd6740. 10.1126/sciadv.abd6740 PMC7904259 33627421

[28] a) T. A. Wynn , K. M. Vannella , Immunity 2016, 44, 450; 26982353 10.1016/j.immuni.2016.02.015 PMC4794754

[29] Y. Shen , Y. Liu , J. K. Nunes , C. Wang , M. Xu , M. K. T. To , H. A. Stone , H. C. Shum , Adv. Mater. 2023, 35, 2211637. 10.1002/adma.202211637 36789886

[30] A. Rübsam , S. Parikh , P. E. Fort , Int. J. Mol. Sci. 2018, 19, 942. 29565290 10.3390/ijms19040942 PMC5979417

[31] a) P. G. Bowler , B. I. Duerden , D. G. Armstrong , Clin. Microbiol. Rev. 2001, 14, 244; 11292638 10.1128/CMR.14.2.244-269.2001 PMC88973

[32] H. Tian , L. Wang , W. Xie , C. Shen , G. Guo , J. Liu , C. Han , L. Ren , Y. Liang , Y. Tang , Y. Wang , M. Yin , J. Zhang , Y. Huang , Burns Trauma 2018, 6, 14. 29850643 10.1186/s41038-018-0118-z PMC5964711

[33] a) S. Kivity , N. Agmon‐Levin , M. Blank , Y. Shoenfeld , Trends Immunol. 2009, 30, 409; 19643667 10.1016/j.it.2009.05.005

[34] C. Wang , E. Shirzaei Sani , C.‐D. Shih , C. T. Lim , J. Wang , D. G. Armstrong , W. Gao , Nat. Rev. Mater. 2024, 9, 550. 10.1038/s41578-024-00693-y PMC12176411 40535534

[35] a) S. Tavakoli , H. Mokhtari , M. Kharaziha , A. Kermanpur , A. Talebi , J. Moshtaghian , Mater. Sci. Eng. 2020, 111, 110837; 10.1016/j.msec.2020.110837 32279800

[36] a) P. Contessotto , D. Orbanić , M. D. Costa , C. Jin , P. Owens , S. Chantepie , C. Chinello , J. Newell , F. Magni , D. Papy‐Garcia , N. G. Karlsson , M. Kilcoyne , P. Dockery , J. C. Rodríguez‐Cabello , A. Pandit , Sci. Transl. Med. 2021, 13, aaz5380; 10.1126/scitranslmed.aaz5380 33597263

[37] Y. Dong , Y. Zheng , K. Zhang , Y. Yao , L. Wang , X. Li , J. Yu , B. Ding , Adv. Fiber Mater. 2020, 2, 212.

[38] L. P. da Silva , R. L. Reis , V. M. Correlo , A. P. Marques , Annu. Rev. Biomed. Eng. 2019, 21, 145. 30822099 10.1146/annurev-bioeng-060418-052422

[39] a) B. Xu , X. G. Wang , Z. L. Meng , L. Y. Zhu , Y. X. Zhang , P. Wu , C. M. Han , Chin. J. Traumatol. 2023, 26, 187; 37037680 10.1016/j.cjtee.2022.12.008 PMC10388252

[40] H. Yang , Y. Qiao , Z. Chang , H. Deng , P. He , H. Zhou , Adv. Mater. 2020, 32, 2004240. 10.1002/adma.202004240 32797719

[41] S. Gibbs , H. M. Van Den Hoogenband , G. Kirtschig , C. D. Richters , S. W. Spiekstra , M. Breetveld , R. J. Scheper , E. M. De Boer , Br. J. Dermatol. 2006, 155, 267. 16882162 10.1111/j.1365-2133.2006.07266.x

[42] M. A. Towler , E. W. Rush , M. K. Richardson , C. L. Williams , Clin. Podiatr. Med. Surg. 2018, 35, 357. 29861018 10.1016/j.cpm.2018.02.006

[43] R. Liang , R. Li , W. Mo , X. Zhang , J. Ye , C. Xie , W. Li , Z. Peng , Y. Gu , Y. Huang , S. Zhang , X. Wang , H. Ouyang , Bioact. Mater. 2024, 40, 541. 39055734 10.1016/j.bioactmat.2024.06.024 PMC11269296

[44] S. Tan , Z. Liu , M. Cong , X. Zhong , Y. Mao , M. Fan , F. Jiao , H. Qiao , J Control Release 2024, 368, 355. 38432468 10.1016/j.jconrel.2024.02.045

[45] Q. Wu , R. Yang , W. Fan , L. Wang , J. Zhan , T. Cao , Q. Liu , X. Piao , Y. Zhong , W. Zhao , S. Zhang , J. Yu , S. Liang , T. M. Roberts , B. Wang , Z. Liu , Adv. Sci. 2024, 11, 2310162. 10.1002/advs.202310162 PMC11165486 38602439

[46] H. Xie , Z. Wang , R. Wang , Q. Chen , A. Yu , A. Lu , Adv. Funct. Mater. 2024, 34, 2401209.

[47] S. J. Forbes , N. Rosenthal , Nat. Med. 2014, 20, 857. 25100531 10.1038/nm.3653

[48] J. G. Seavey , Z. A. Masters , G. C. Balazs , S. M. Tintle , J. Sabino , M. E. Fleming , I. L. Valerio , Regen. Med. 2016, 11, 81. 26681342 10.2217/rme.15.83

[49] G. Nicoletti , F. Brenta , M. Bleve , T. Pellegatta , A. Malovini , A. Faga , P. Perugini , J. Tissue Eng. Regen. Med. 2015, 9, 460. 24962375 10.1002/term.1939

[50] D. Zhao , Z. Yu , Y. Li , Y. Wang , Q. Li , D. Han , J. Mol. Histol. 2020, 51, 251. 32388839 10.1007/s10735-020-09877-6

[51] H. Xia , Z. Dong , Q. Tang , R. Ding , Y. Bai , K. Zhou , L. Wu , L. Hao , Y. He , J. Yang , H. Mao , Z. Gu , Adv. Funct. Mater. 2023, 33, 2215116.

[52] S. Liu , Y. Zhao , M. Li , L. Nie , Q. Wei , O. V. Okoro , H. Jafari , S. Wang , J. Deng , J. Chen , A. Shavandi , L. Fan , Chem. Eng. J. 2023, 466, 143016.

[53] Q. Guo , T. Yin , W. Huang , R. Nan , T. Xiang , S. Zhou , Adv. Healthcare Mater. 2024, 13, e2304536. 10.1002/adhm.202304536 38519046

[54] a) G. Zhong , P. Lei , P. Guo , Q. Yang , Y. Duan , J. Zhang , M. Qiu , K. Gou , C. Zhang , Y. Qu , R. Zeng , Small 2024, 20, e2309568; 38461520 10.1002/smll.202309568

[55] C. D. Weller , V. Team , G. Sussman , Front. Pharmacol. 2020, 11, 155. 32180720 10.3389/fphar.2020.00155 PMC7059819

[56] L. I. Moura , A. M. Dias , E. Carvalho , H. C. de Sousa , Acta Biomater. 2013, 9, 7093. 23542233 10.1016/j.actbio.2013.03.033

[57] N. Golafshan , R. Rezahasani , M. Tarkesh Esfahani , M. Kharaziha , S. N. Khorasani , Carbohydr. Polym. 2017, 176, 392. 28927623 10.1016/j.carbpol.2017.08.070

[58] R. Thakur , G. Laverty , R. Donnelly , Hydrogels: Design, Synthesis and Application in Drug Delivery and Regenerative Medicine, CRC Press/ Taylor & Francis Group, Boca Raton, FL 2018.

[59] P. Mostafalu , A. Tamayol , R. Rahimi , M. Ochoa , A. Khalilpour , G. Kiaee , I. K. Yazdi , S. Bagherifard , M. R. Dokmeci , B. Ziaie , S. R. Sonkusale , A. Khademhosseini , Small 2018, 14, e1703509. 10.1002/smll.201703509 29978547

[60] J. Zhang , Y. Zheng , J. Lee , J. Hua , S. Li , A. Panchamukhi , J. Yue , X. Gou , Z. Xia , L. Zhu , X. Wu , Nat. Commun. 2021, 12, 1670. 33723267 10.1038/s41467-021-21964-0 PMC7960722

[61] M. Li , X. Liu , L. Tan , Z. Cui , X. Yang , Z. Li , Y. Zheng , K. W. K. Yeung , P. K. Chu , S. Wu , Biomater. Sci. 2018, 6, 2110. 29882566 10.1039/c8bm00499d

[62] Y. Lv , F. Cai , Y. He , L. Li , Y. Huang , J. Yang , Y. Zheng , X. Shi , Acta Biomater. 2023, 159, 95. 36736644 10.1016/j.actbio.2023.01.045

[63] W. Wang , Y. Cui , X. Wei , Y. Zang , X. Chen , L. Cheng , X. Wang , ACS Nano 2024, 18, 15845. 38832685 10.1021/acsnano.4c02825

[64] T. Guan , J. Li , C. Chen , Y. Liu , Adv. Sci. 2022, 9, e2104165. 10.1002/advs.202104165 PMC8981472 35142093

[65] H. Yang , C. Lai , C. Xuan , M. Chai , X. Liu , Y. Chen , X. Shi , Chem. Eng. J. 2020, 398, 125617.

[66] Z. Chen , L. Wang , C. Guo , M. Qiu , L. Cheng , K. Chen , J. Qi , L. Deng , C. He , X. Li , Y. Yan , Acta Biomater. 2023, 155, 218. 36396041 10.1016/j.actbio.2022.11.002

[67] M. Chen , J. Tian , Y. Liu , H. Cao , R. Li , J. Wang , J. Wu , Q. Zhang , Chem. Eng. J. 2019, 373, 413.

[68] P. Rousselle , F. Braye , G. Dayan , Adv. Drug Deliv. Rev. 2019, 146, 344. 29981800 10.1016/j.addr.2018.06.019

[69] S. Yamakawa , K. Hayashida , Burns Trauma 2019, 7, 10. 30993143 10.1186/s41038-019-0148-1 PMC6450003

[70] Y. Brudno , A. B. Ennett‐Shepard , R. R. Chen , M. Aizenberg , D. J. Mooney , Biomaterials 2013, 34, 9201. 23972477 10.1016/j.biomaterials.2013.08.007 PMC3811005

[71] Z. Wang , X. Dong , S. Zhou , Z. Xie , Z. Zalevsky , NPG Asia Mater. 2021, 13, 5.

[72] Y. Niu , Q. Li , Y. Ding , L. Dong , C. Wang , Adv. Drug Deliv. Rev. 2019, 146, 190. 29879493 10.1016/j.addr.2018.06.002

[73] S. Bagherifard , A. Tamayol , P. Mostafalu , M. Akbari , M. Comotto , N. Annabi , M. Ghaderi , S. Sonkusale , M. R. Dokmeci , A. Khademhosseini , Adv. Healthcare Mater. 2016, 5, 175. 10.1002/adhm.201500357 26501166

[74] H. Derakhshandeh , F. Aghabaglou , A. McCarthy , A. Mostafavi , C. Wiseman , Z. Bonick , I. Ghanavati , S. Harris , C. Kreikemeier‐Bower , S. M. M. Basri , J. Rosenbohm , R. Yang , P. Mostafalu , D. Orgill , A. Tamayol , Adv. Funct. Mater. 2020, 30, 1905544. 34354556 10.1002/adfm.201905544 PMC8336080

[75] T. E. Sutherland , D. P. Dyer , J. E. Allen , Science 2023, 379, eabp8964. 36795835 10.1126/science.abp8964

[76] E. A. Lester , J. E. Babensee , J. Biomed. Mater. Res. 2003, 64A, 397. 10.1002/jbm.a.10378 12870471

[77] a) Y.‐Z. Lu , B. Nayer , S. K. Singh , Y. K. Alshoubaki , E. Yuan , A. J. Park , K. Maruyama , S. Akira , M. M. Martino , Nature 2024, 628, 604; 38538784 10.1038/s41586-024-07237-y PMC11023938

[78] S. Butenko , R. R. Nagalla , C. F. Guerrero‐Juarez , F. Palomba , L.‐M. David , R. Q. Nguyen , D. Gay , A. A. Almet , M. A. Digman , Q. Nie , P. O. Scumpia , M. V. Plikus , W. F. Liu , Nat. Commun. 2024, 15, 6820. 39122702 10.1038/s41467-024-50072-y PMC11315930

[79] O. A. Peña , P. Martin , Nat. Rev. Mol. Cell Biol. 2024, 25, 599. 38528155 10.1038/s41580-024-00715-1

[80] a) P. J. Murray , Annu. Rev. Physiol. 2017, 79, 541; 27813830 10.1146/annurev-physiol-022516-034339

[81] a) S. Willenborg , L. Injarabian , S. A. Eming , Cold Spring Harb. Perspect. Biol. 2022, 14, a041216; 36041784 10.1101/cshperspect.a041216 PMC9732901

[82] M. Horckmans , L. Ring , J. Duchene , D. Santovito , M. J. Schloss , M. Drechsler , C. Weber , O. Soehnlein , S. Steffens , Eur. Heart J. 2017, 38, ehw002. 10.1093/eurheartj/ehw002 28158426

[83] B. D. Levy , C. B. Clish , B. Schmidt , K. Gronert , C. N. Serhan , Nat. Immunol. 2001, 2, 612. 11429545 10.1038/89759

[84] C. N. Serhan , J. Savill , Nat. Immunol. 2005, 6, 1191. 16369558 10.1038/ni1276

[85] a) J. F. Maddox , M. Hachicha , T. Takano , N. A. Petasis , V. V. Fokin , C. N. Serhan , J. Biol. Chem. 1997, 272, 6972; 9054386 10.1074/jbc.272.11.6972

[86] T. Lucas , A. Waisman , R. Ranjan , J. Roes , T. Krieg , W. Müller , A. Roers , S. A. Eming , J. Immunol. 2010, 184, 3964. 20176743 10.4049/jimmunol.0903356

[87] B.‐H. Cha , S. R. Shin , J. Leijten , Y.‐C. Li , S. Singh , J. C. Liu , N. Annabi , R. Abdi , M. R. Dokmeci , N. E. Vrana , A. M. Ghaemmaghami , A. Khademhosseini , Adv. Healthcare Mater 2017, 6, 1700289. 10.1002/adhm.201700289 PMC5677560 28782184

[88] S. Franz , S. Rammelt , D. Scharnweber , J. C. Simon , Biomaterials 2011, 32, 6692. 21715002 10.1016/j.biomaterials.2011.05.078

[89] S. Mizrahy , S. R. Raz , M. Hasgaard , H. Liu , N. Soffer‐Tsur , K. Cohen , R. Dvash , D. Landsman‐Milo , M. G. Bremer , S. M. Moghimi , D. Peer , J. Control Release 2011, 156, 231. 21745506 10.1016/j.jconrel.2011.06.031

[90] F. Zamboni , S. Vieira , R. L. Reis , J. Miguel Oliveira , M. N. Collins , Prog. Mater. Sci. 2018, 97, 97.

[91] X. Cui , R. T. Morales , W. Qian , H. Wang , J. P. Gagner , I. Dolgalev , D. Placantonakis , D. Zagzag , L. Cimmino , M. Snuderl , R. H. W. Lam , W. Chen , Biomaterials 2018, 161, 164. 29421553 10.1016/j.biomaterials.2018.01.053 PMC8059366

[92] a) T. Yuan , L. Zhang , L. Feng , H. Fan , X. Zhang , Biotechnol. Prog. 2010, 26, 1749; 20865774 10.1002/btpr.484

[93] A. R. Donaldson , C. E. Tanase , D. Awuah , P. Vasanthi Bathrinarayanan , L. Hall , M. Nikkhah , A. Khademhosseini , F. Rose , C. Alexander , A. M. Ghaemmaghami , Front. Bioeng. Biotechnol. 2018, 6, 116. 30283776 10.3389/fbioe.2018.00116 PMC6156527

[94] G. Fingerle , A. Pforte , B. Passlick , M. Blumenstein , M. Ströbel , H. W. Ziegler‐Heitbrock , Blood 1993, 82, 3170. 7693040

[95] N. Mokarram , R. V. Bellamkonda , Ann. Biomed. Eng. 2014, 42, 338. 24297492 10.1007/s10439-013-0941-0

[96] J. Y. Hsieh , T. D. Smith , V. S. Meli , T. N. Tran , E. L. Botvinick , W. F. Liu , Acta Biomater. 2017, 47, 14. 27662809 10.1016/j.actbio.2016.09.024 PMC5426227

[97] B. Chancheewa , S. Buranapraditkun , C. Laomeephol , P. Rerknimitr , S. Kanokpanont , S. Damrongsakkul , J. Klaewsongkram , Mater. Today Commun. 2020, 24, 101044.

[98] a) Y. R. Park , M. T. Sultan , H. J. Park , J. M. Lee , H. W. Ju , O. J. Lee , D. J. Lee , D. L. Kaplan , C. H. Park , Acta Biomater. 2018, 67, 183; 29242162 10.1016/j.actbio.2017.12.006

[99] D. Chouhan , T. U. Lohe , P. K. Samudrala , B. B. Mandal , Adv. Healthcare Mater. 2018, 7, e1801092. 10.1002/adhm.201801092 30379407

[100] S. He , Z. Li , L. Wang , N. Yao , H. Wen , H. Yuan , J. Zhang , Z. Li , C. Shen , Bioact. Mater. 2024, 35, 17. 38304915 10.1016/j.bioactmat.2024.01.005 PMC10831190

[101] N. Li , X. Lu , Y. Yang , S. Ning , Y. Tian , M. Zhou , Z. Wang , L. Wang , J. Zang , Adv. Healthcare Mater. 2024, 13, e2303314. 10.1002/adhm.202303314 38558386

[102] a) A. Curtis , C. Wilkinson , Biomaterials 1997, 18, 1573; 9613804 10.1016/s0142-9612(97)00144-0

[103] J. Zhang , Y. Zhu , J. Song , J. Yang , C. Pan , T. Xu , L. Zhang , ACS Appl. Mater. Interfaces 2018, 10, 6879. 29393622 10.1021/acsami.7b17670

[104] T. L. Lopez‐Silva , D. G. Leach , A. Azares , I. C. Li , D. G. Woodside , J. D. Hartgerink , Biomaterials 2020, 231, 119667. 31855625 10.1016/j.biomaterials.2019.119667 PMC7049098

[105] S. Singh , D. Awuah , H. M. Rostam , R. D. Emes , N. K. Kandola , D. Onion , S. S. Htwe , B. Rajchagool , B. H. Cha , D. Kim , P. J. Tighe , N. E. Vrana , A. Khademhosseini , A. Ghaemmaghami , ACS Biomater. Sci. Eng. 2017, 3, 969. 33429569 10.1021/acsbiomaterials.7b00104

[106] L. Zhang , Z. Cao , T. Bai , L. Carr , J. R. Ella‐Menye , C. Irvin , B. D. Ratner , S. Jiang , Nat. Biotechnol. 2013, 31, 553. 23666011 10.1038/nbt.2580

[107] Z. Li , K. M. Bratlie , ACS Appl. Bio Mater. 2019, 2, 217. 10.1021/acsabm.8b00562 35016344

[108] Y. Li , R. J. Lee , K. Yu , Y. Bi , Y. Qi , Y. Sun , Y. Li , J. Xie , L. Teng , ACS Appl. Mater. Interfaces 2016, 8, 26613. 27617513 10.1021/acsami.6b09991

[109] R. Sridharan , B. Cavanagh , A. R. Cameron , D. J. Kelly , F. J. O'Brien , Acta Biomater. 2019, 89, 47. 30826478 10.1016/j.actbio.2019.02.048

[110] Z. Zhuang , Y. Zhang , S. Sun , Q. Li , K. Chen , C. An , L. Wang , J. van den Beucken , H. Wang , ACS Biomater. Sci. Eng. 2020, 6, 3091. 33463297 10.1021/acsbiomaterials.0c00295

[111] Y. Liu , A. Suarez‐Arnedo , L. Riley , T. Miley , J. Xia , T. Segura , Adv. Healthcare Mater. 2023, 12, e2300823. 10.1002/adhm.202300823 PMC10592513 37165945

[112] L. R. Madden , D. J. Mortisen , E. M. Sussman , S. K. Dupras , J. A. Fugate , J. L. Cuy , K. D. Hauch , M. A. Laflamme , C. E. Murry , B. D. Ratner , Proc. Natl. Acad. Sci. U S A 2010, 107, 15211. 20696917 10.1073/pnas.1006442107 PMC2930533

[113] M. Gnecchi , H. He , O. D. Liang , L. G. Melo , F. Morello , H. Mu , N. Noiseux , L. Zhang , R. E. Pratt , J. S. Ingwall , V. J. Dzau , Nat. Med. 2005, 11, 367. 15812508 10.1038/nm0405-367

[114] S. S. Iyer , G. Cheng , Crit. Rev. Immunol. 2012, 32, 23. 22428854 10.1615/critrevimmunol.v32.i1.30 PMC3410706

[115] M. Brennan , P. Layrolle , D. J. Mooney , Adv. Funct. Mater. 2020, 30, 1909125. 32952493 10.1002/adfm.201909125 PMC7494127

[116] J. Maggini , G. Mirkin , I. Bognanni , J. Holmberg , I. M. Piazzón , I. Nepomnaschy , H. Costa , C. Cañones , S. Raiden , M. Vermeulen , J. R. Geffner , PLoS One 2010, 5, e9252. 20169081 10.1371/journal.pone.0009252 PMC2821929

[117] a) A. B. Aurora , E. N. Olson , Cell Stem Cell 2014, 15, 14; 24996166 10.1016/j.stem.2014.06.009 PMC4131296

[118] R. Blázquez , F. M. Sánchez‐Margallo , V. Álvarez , A. Usón , J. G. Casado , Acta Biomater. 2016, 31, 221. 26654766 10.1016/j.actbio.2015.11.057

[119] K. H. Vining , A. Stafford , D. J. Mooney , Biomaterials 2019, 188, 187. 30366219 10.1016/j.biomaterials.2018.10.013 PMC6279497

[120] A. D. Guerra , W. E. Rose , P. Hematti , W. J. Kao , Acta Biomater. 2017, 51, 184. 28069512 10.1016/j.actbio.2017.01.021 PMC5704963

[121] C. Wang , M. Wang , T. Xu , X. Zhang , C. Lin , W. Gao , H. Xu , B. Lei , C. Mao , Theranostics 2019, 9, 65. 30662554 10.7150/thno.29766 PMC6332800

[122] Q. Xu , S. A. , Y. Gao , L. Guo , J. Creagh‐Flynn , D. Zhou , U. Greiser , Y. Dong , F. Wang , H. Tai , W. Liu , W. Wang , W. Wang , Acta Biomater. 2018, 75, 63. 29803782 10.1016/j.actbio.2018.05.039

[123] J. R. García , M. Quirós , W. M. Han , M. N. O'Leary , G. N. Cox , A. Nusrat , A. J. García , Biomaterials 2019, 220, 119403. 31401468 10.1016/j.biomaterials.2019.119403 PMC6717550

[124] Y. Dong , M. Cui , J. Qu , X. Wang , S. H. Kwon , J. Barrera , N. Elvassore , G. C. Gurtner , Acta Biomater. 2020, 108, 56. 32251786 10.1016/j.actbio.2020.03.040

[125] A. Singh , M. Talekar , A. Raikar , M. Amiji , J Control Release 2014, 190, 515. 24747762 10.1016/j.jconrel.2014.04.021

[126] N. G. Frangogiannis , J. Thorac. Dis. 2017, 9, S52. 28446968 10.21037/jtd.2016.11.19 PMC5383562

[127] E. Gounaris , N. R. Blatner , K. Dennis , F. Magnusson , M. F. Gurish , T. B. Strom , P. Beckhove , F. Gounari , K. Khazaie , Cancer Res. 2009, 69, 5490. 19570783 10.1158/0008-5472.CAN-09-0304 PMC2881579

[128] a) C. Bogdan , Y. Vodovotz , C. Nathan , J. Exp. Med. 1991, 174, 1549; 1744584 10.1084/jem.174.6.1549 PMC2119047

[129] E. Nova‐Lamperti , G. Fanelli , P. D. Becker , P. Chana , R. Elgueta , P. C. Dodd , G. M. Lord , G. Lombardi , M. P. Hernandez‐Fuentes , Sci. Rep. 2016, 6, 20044. 26795594 10.1038/srep20044 PMC4726240

[130] a) Y. Guo , Y. Q. Xie , M. Gao , Y. Zhao , F. Franco , M. Wenes , I. Siddiqui , A. Bevilacqua , H. Wang , H. Yang , B. Feng , X. Xie , C. M. Sabatel , B. Tschumi , A. Chaiboonchoe , Y. Wang , W. Li , W. Xiao , W. Held , P. Romero , P. C. Ho , L. Tang , Nat. Immunol. 2021, 22, 746; 34031618 10.1038/s41590-021-00940-2 PMC7610876

[131] P. J. Murray , T. A. Wynn , Nat. Rev. Immunol. 2011, 11, 723. 21997792 10.1038/nri3073 PMC3422549

[132] V. A. Kumar , N. L. Taylor , S. Shi , N. C. Wickremasinghe , R. N. D'Souza , J. D. Hartgerink , Biomaterials 2015, 52, 71. 25818414 10.1016/j.biomaterials.2015.01.079 PMC4613801

[133] M. F. Shamji , P. Hwang , R. W. Bullock , S. B. Adams , D. L. Nettles , L. A. Setton , J. Biomed. Mater. Res. B Appl. Biomater. 2009, 90, 319. 19072988 10.1002/jbm.b.31289 PMC2775925

[134] J. A. Kaplan , P. Barthélémy , M. W. Grinstaff , Chem. Commun. 2016, 52, 5860. 10.1039/c6cc02221a 27049283

[135] E. E. Friedrich , L. T. Sun , S. Natesan , D. O. Zamora , R. J. Christy , N. R. Washburn , J. Biomed. Mater. Res. A 2014, 102, 1527. 23765644 10.1002/jbm.a.34829 PMC4119967

[136] D. P. Vasconcelos , M. Costa , I. F. Amaral , M. A. Barbosa , A. P. Águas , J. N. Barbosa , Biomaterials 2015, 37, 116. 25453942 10.1016/j.biomaterials.2014.10.035

[137] M. Puertas‐Bartolomé , L. Benito‐Garzón , S. Fung , J. Kohn , B. Vázquez‐Lasa , J. San Román , Mater. Sci. Eng. C Mater. Biol. Appl. 2019, 105, 110040. 31546368 10.1016/j.msec.2019.110040

[138] C. S. Verbeke , S. Gordo , D. A. Schubert , S. A. Lewin , R. M. Desai , J. Dobbins , K. W. Wucherpfennig , D. J. Mooney , Adv. Healthcare Mater. 2017, 6, 1600773. 10.1002/adhm.201600773 PMC5518671 28116870

[139] R. E. Mirza , M. M. Fang , W. J. Ennis , T. J. Koh , Diabetes 2013, 62, 2579. 23493576 10.2337/db12-1450 PMC3712034

[140] S. M. Huang , C. S. Wu , M. H. Chiu , C. H. Wu , Y. T. Chang , G. S. Chen , C. E. Lan , J. Dermatol. Sci. 2019, 96, 159. 31761388 10.1016/j.jdermsci.2019.11.004

[141] T. Horiuchi , H. Mitoma , S. Harashima , H. Tsukamoto , T. Shimoda , Rheumatology (Oxford) 2010, 49, 1215. 20194223 10.1093/rheumatology/keq031 PMC2886310

[142] X. Wu , W. Xu , X. Feng , Y. He , X. Liu , Y. Gao , S. Yang , Z. Shao , C. Yang , Z. Ye , Int. J. Immunopathol. Pharmacol. 2015, 28, 351. 26197804 10.1177/0394632015593228

[143] M. M. Alvarez , J. C. Liu , G. Trujillo‐de Santiago , B. H. Cha , A. Vishwakarma , A. M. Ghaemmaghami , A. Khademhosseini , J. Control Release 2016, 240, 349. 26778695 10.1016/j.jconrel.2016.01.026 PMC4945478

[144] S. Liu , M. Zhao , Y. Zhou , L. Li , C. Wang , Y. Yuan , L. Li , G. Liao , W. Bresette , Y. Chen , J. Cheng , Y. Lu , J. Liu , Acta Biomater. 2020, 103, 102. 31843715 10.1016/j.actbio.2019.12.011

[145] a) Y. Wu , Z. Yang , K. Cheng , H. Bi , J. Chen , Acta Pharm. Sin. B 2022, 12, 4287; 36562003 10.1016/j.apsb.2022.11.007 PMC9764074

[146] I. C. Boothby , J. N. Cohen , M. D. Rosenblum , Sci. Immunol. 2020, 5, aaz9631. 10.1126/sciimmunol.aaz9631 PMC7274208 32358172

[147] L. A. Kalekar , J. N. Cohen , N. Prevel , P. M. Sandoval , A. N. Mathur , J. M. Moreau , M. M. Lowe , A. Nosbaum , P. J. Wolters , A. Haemel , F. Boin , M. D. Rosenblum , Sci. Immunol. 2019, 4, aaw2910. 10.1126/sciimmunol.aaw2910 PMC6848056 31492709

[148] a) P. A. Szabo , M. Miron , D. L. Farber , Sci. Immunol. 2019, 4, aas9673; 10.1126/sciimmunol.aas9673 PMC6778482 30952804

[149] M. H. Jang , N. P. Seth , K. W. Wucherpfennig , J. Immunol. 2003, 171, 4175. 14530340 10.4049/jimmunol.171.8.4175

[150] M. E. Ogle , J. R. Krieger , L. E. Tellier , J. McFaline‐Figueroa , J. S. Temenoff , E. A. Botchwey , ACS Biomater. Sci. Eng. 2018, 4, 1241. 29682605 10.1021/acsbiomaterials.6b00706 PMC5909722

